# The trypanosome vault particle is composed of multiple major vault protein paralogs and harbors vault RNA

**DOI:** 10.1016/j.jbc.2025.110706

**Published:** 2025-09-11

**Authors:** Anna Zavrelova, Siqi Shen, Farnaz Zahedifard, Emmanuel Ayodeji Agbebi, Silke Braune, Susanne Kramer, Martin Zoltner

**Affiliations:** 1 Department of Parasitology, Faculty of Science, Charles University in Prague, Biocev, Vestec, Czech Republic; 2 Biocenter, University of Würzburg, Würzburg, Germany

**Keywords:** major vault protein, vault particle, vault RNA, BioID, evolution, affinity isolation, cryomilling, trypanosoma

## Abstract

Many but not all Eukaryotes have protein-enclosed compartments called vaults. Vaults are composed of multiple copies of the major vault protein, symmetrically assembled into a basket-like shell. A human cell contains approximately 100,000 vault particles, the vast majority localized to the cytosol but also observed in the nucleus and at the nuclear pore complex. Whilst there is intriguing structural information of the vault shell, the function of vaults remains largely elusive, apart from a potential contribution to mRNA maturation. We set out to explore the vault interactome in the early branching eukaryote *Trypanosoma brucei* employing a combination of affinity capture and TurboID proximity labelling. *T. brucei* encodes three major vault protein (MVP) paralogs, which exhibit a considerable degree of divergence. Unexpectedly, affinity capture proteomics with one MVP as a bait precipitated the other two paralogs, detected with similar intensities, indicating the possibility that all three are incorporated into the same particle. Dual color fluorescence microscopy of MVP pairs fused with different GFP-variants confirmed that all three paralogs are incorporated into a single vault shell. Our combined interactome data, including immune-isolations with varying stringencies, suggest a vault particle core composition of three MVPs homologs and the telomerase-associated protein 1 (TEP1), which has been described as a vault component in various organisms. Further, we demonstrate the association of vtRNA with the particle and suggest a cohort of potential transient vault interactors, dominated by RNA-binding proteins and splicing factors, which were found enriched in both orthogonal interactome approaches.

Vaults are large oligomeric ribonucleoprotein (RNP) complexes present in many eukaryotic organisms. With dimensions of approximately 670 × 400 A, roughly equivalent to the size of three ribosomes, vaults are likely the largest RNP particle structure in eukaryotic cells (1). The rat vault particle consists of two equivalent halves each comprising 39 identical major vault proteins (MVPs) 104 kDa chains (1), associating into a basket-like shell with two protruding caps and an invaginated waist at the junction (Fig. 1*A*). The name of vaults comes from the complex structure of their shell reminiscent of the vaulted ceilings in cathedrals (2).

**Figure 1:**
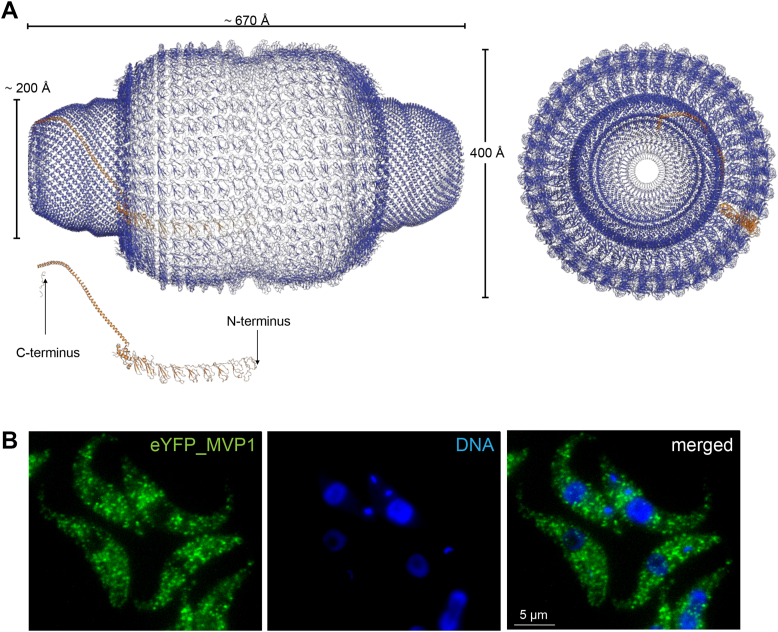
**MVP1 forms cytoplasmic particles.***A*, the mammalian vault shell assembly consists of 78 MVP subunits as illustrated by the cartoon representation of the X-ray structure of the rat liver vault particle (pdb ID: 4V60) (1), shown in side view (*left*) and top-view (*right*) with approximate dimensions indicated. One representative MVP monomer is colored orange and drawn as isolated cartoon with N- and C-terminus indicated. *B*, *T. brucei* MVP1 (Tb927.5.4660) with N-terminal eYFP fusion assembles to particles localized in the cytoplasm. Shown are single plane raw images for eYFP (*green*) and DAPI (*blue*) fluorescence and a respective merge. The observed dots from direct eYFP fluorescence appear as equally sized particles (>100 per cell) evenly distributed across the cytoplasm.

Vaults are widely present in eukaryotic phyla albeit with patchy distribution: they are present in diverse species such as sea urchins (3), slime mold (4), electric ray (5), and mammals (2) but surprisingly absent in plant cells and many model organisms like *Saccharomyces cerevisiae*, *Caenorhabditis elegans* or *Drosophila melanogaster* (6). Phylogenetic analysis suggests the presence of vaults in the last eukaryote common ancestor (LECA) (7), with subsequent loss in several clades including insects, fungi, nematodes and plants.

Mammalian vaults also contain two minor proteins (8), the 193 kDa vault poly-adenosine diphosphate–ribose polymerase (vPARP) with a function in substrate ADP ribosylation (9) and the 240 kDa telomerase-associated protein 1 (TEP1) (10), which forms a RNP complex with small untranslated RNAs, termed vault RNA (vtRNA) (11). vtRNA association with TEP1 is important for their stability (12, 13). TEP1 was initially discovered to interact with the telomerase RNP (14), but a knockout in mice failed to evoke any significant effect on telomere maintenance (15). The MVP forms the outer shell of the vault, and the two minor proteins are probably localized at the caps (16).

The morphology and structure of vaults are highly conserved among diverse eukaryotic species (8, 17), which would implicate a significant biological function. However, unexpectedly, MVP knockout in mice failed to evoke significant phenotypic consequences (18, 19). Likewise, loss of TEP1 did neither affect mice's health nor their ageing (12, 15). Altogether, there is a plethora of functional leads that remain inconclusive: Vaults have been linked to infection resistance (20), cellular signaling pathways (6), roles in immune response (20, 21), and autophagy (22). Similarly, the role of vtRNA, shown to be dispensable for normal mammalian development and viability (23), remains enigmatic.

A recent landmark study established a role of vtRNA in regulating mRNA metabolism (13) in *Trypanosoma brucei*, a unicellular parasitic organism that causes human African trypanosomiasis and the related veterinary disease Nagana in cattle. *T*. *brucei* belongs to the Kinetoplastida within the Discoba lineage, likely representing an early branch following eukaryogenesis, and has become a model system for evolutionary cell biology, combining tractability, relevant phylogenetic position, divergent biology, and comparative simplicity in genome and proteome size. Trypanosomes transcribe all mRNAs co-transcriptionally as long polycistrons and process these precursors by spliced leader (SL)- RNA-mediated trans-splicing (24), which is therefore an essential process in *T. brucei* gene expression. The demonstration that downregulation of vtRNA levels in trypanosomes impacts the production of trans-spliced mRNA (13) sparked our curiosity about a potential involvement of the vault particle in this process, as *T. brucei* encodes MVP proteins.

Hence, we set out to explore the MVP interactome of *T. brucei*, employing a combination of proximity-labeling and cryomilling affinity capture proteomics, with both these approaches capable of detecting transient interactions (25, 26).

Our combined interactome data suggest interaction of the vault particle with components of the splicing machinery, consistent with the observed function of trypanosome vtRNA (13). Further, we provide evidence that the vault shell in trypanosomes is composed of three surprisingly divergent paralogs of the major vault protein, which appears to be a common characteristic in Kinetoplastida. Lastly, we localize and quantify the single vtRNA and show a dominant cytoplasmic localization associated with the vault particle at an abundance similar to the most abundant trypanosome mRNA.

## Results and discussion

### Trypanosomes encode three MVP paralogs

We detected three MVP paralogs in the *T. brucei* genome that we designated MVP1 (Tb927.5.4460), MVP2 (Tb927.10.1990), and MVP3 (Tb927.10.6310). These three paralogs exhibit surprising divergence: MVP1 shares 58% and 56% sequence similarity with MVP2 and MVP3, respectively, while the latter two are even less related (52% sequence similarity). Notably, MVP1 shares significantly higher sequence similarity with the mammalian MVP (67% for the rat MVP (Q62667)) than with its paralogs (for a multiple sequence alignment see Fig. S1).

### MVP1 N-terminal eYFP fusion localizes to cytoplasmic particles

MVP1 was endogenously tagged with eYFP at both the N- and the C-terminus in procyclic form (PCF) *T. brucei,* and resulting clonal cell lines were analyzed by western blotting. C-terminal tagging led to truncated fusion proteins at lower molecular weight (data not shown), in accordance with observations for the mammalian MVP tolerating N-terminal, but not C-terminal fusion tags (27). The N-terminal MVP1 eYFP fusion protein migrated at the expected size (Fig. S2). Inspection of ^eYFP^MVP1-expressing cells by fluorescence microscopy, monitoring direct eYFP fluorescence, revealed the formation of equally sized, small particles, sometimes elongated, localizing to the cytoplasm but not the nucleus (Fig. 1), in an even distribution that did not change during cell cycle stages (Fig. S3). Similar localizations were observed for MVP2 and MVP3 (see below). In the genome-wide localization project TrypTag (28), C-terminal tagging of MVP1 and MVP2 had failed, but for MVP3, a similar localization to cytoplasmic points has been observed. N-terminal tagging led to a patchy cytoplasmic signal for MVP1, and cytoplasmic points were observed for MVP2 and MVP3, resembling our imaging results.

The detected points are likely individual vault particles, with the eYFP of the multiple subunits acting as an enhancer of the fluorescence, enabling the visualization of single particles despite their small size of only 70 nm. Formally, aggregations of multiple vault particles to a larger particle cannot be excluded, albeit such aggregations would be expected to be more variable in size. The vault particles are highly abundant, too abundant for accurate counting, similar to the situation in (the much larger) mammalian cells that are estimated to contain 10,000 vault particles.

### Affinity capture of MVP1

**Figure 2:**
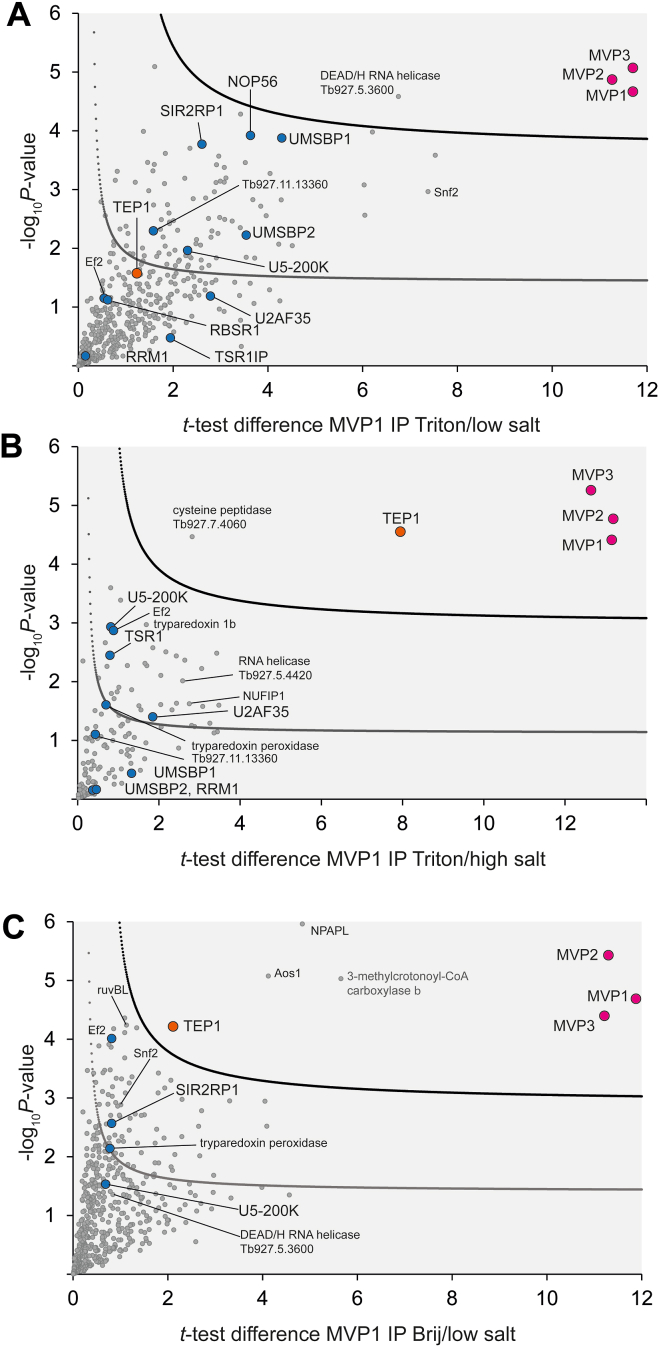
**^eYFP^MVP1 affinity capture** under low salt extraction conditions (A) isolates both MVP paralogs and enriches components associated with splicing, replication, chromatin remodeling and DNA repair. Volcano plots for statistical analysis of cryomilling affinity capture experiments. To generate the volcano plots, the −log_10_*p*-value was plotted *versus* the *t* test difference, comparing each respective bait experiment to the parental control. Potential interactors were classified according to their position in the plot, applying statistical cut-off curves SigA and SigB (see Methods section). Selected protein groups are labeled and colored (purple for MVPs; orange for TEP1; blue for candidate interactors that were also found under more stringent extraction conditions (*B* and *C*) and orthogonal experiments (Fig. 3). RuvBL1 (Tb927.4.1270); RuvBL2 (Tb927.4.2000); SWI/SNF-related helicase (Tb927.11.10730); Snf2 (Tb927.3.5440).

To determine the vault interactome, we subjected ^eYFP^MVP1-expressing cells to cryomilling affinity capture proteomics. Cryomilling allowed rapid disruption of cells under near native conditions (29), and subsequently, the MVP1 complex was captured using anti-GFP single-chain antibodies for immune-isolation. Two detergent conditions were initially evaluated for the MVP1 isolation, along with parental controls, and captured proteins were visualized using silver-stain SDS-PAGE, allowing identification of conditions delivering optimal signal to background ratio (Fig. S4). Based on this analysis, we chose a buffer containing 0.1% Triton X-100 for interactome analysis. Captured complexes were analyzed using liquid chromatography coupled to tandem mass spectrometry (LC-MSMS) and compared with a parallel affinity capture from untagged parental cells under identical conditions (Fig. 2*A*). All analyses were performed in at least triplicate and analyzed by label-free quantification (LFQ) using MaxQuant and Perseus (25).

As the observed interactome was unexpectedly large and the prototypic vault interactor TEP1 was only mildly enriched, additional, more stringent extraction conditions were applied. First, we used the same Triton buffer but increased the salt concentration to 0.5 M NaCl. Secondly, we substituted the detergent for BriJ36 (Fig. S4). In both conditions, we obtained significant TEP1 enrichment (Fig. 2, *B* and *C*). The cohort of proteins with a role in DNA repair was absent in the high-salt and smaller in the BriJ36 experiment, but the helicase SNF2, engaging in chromatin remodeling (30), was still found enriched in the latter condition. To interrogate a potential role of MVP1 in DNA-repair, we challenged bloodstream form (BSF) *T. brucei* with cisplatin, a DNA-damaging, lesion-inducing compound (31), which has been previously applied to study DNA-repair in *T. brucei* (32). Monitoring viability, we could not detect a significant shift in cisplatin sensitivity between MVP1 RNAi-silenced and parental cells (Fig. S5).

### Proximity labeling with MVP1 and TEP1

With affinity capture producing considerable disagreement over the different extraction conditions, we chose to employ an orthogonal interactome approach to validate candidate interactors: TurboID, a biotin ligase mutant, labelling lysine residues in close proximity (33). MVP1 was fused to an N-terminal TurboID-2xHA tandem tag.

As a further bait for proximity labelling, we selected TEP1, the canonical vault particle component, which was detected in the affinity capture approach but appeared to be underrepresented in the Triton/low-salt extraction.

**Figure 3:**
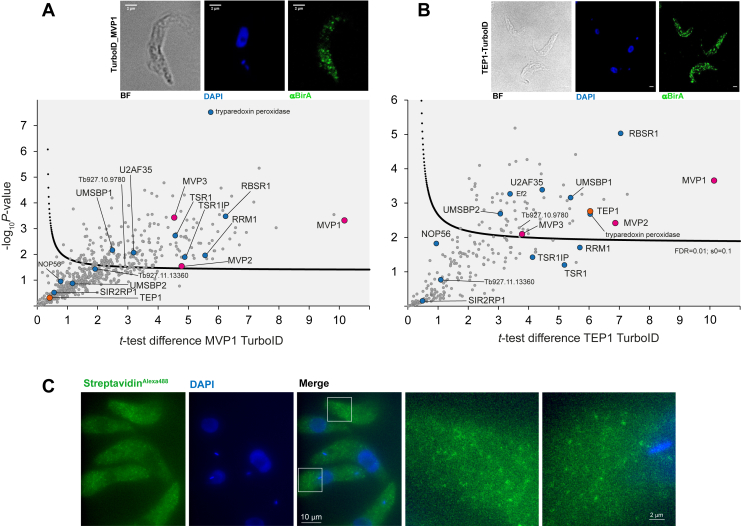
**MVP1 and TEP1 TurboID.** Immunofluorescence detection of MVP1 (*A*) and TEP1 (*B*) TurboID baits and respective volcano plots for statistical analysis of TurboID experiments. To generate the volcano plots, the − log_10_*p*-value was plotted *versus* the *t* test difference, comparing each respective bait experiment to the parental control. Potential interactors were classified according to their position in the plot, applying statistical cut-off curves, of which SigA is drawn (see Methods section). Selected protein groups are labeled and colored (purple for MVPs; orange for TEP1; blue for candidate interactors that were also found enriched in affinity capture. *C*, TurboID-2xHA-TEP1 was detected with streptavidin-Alexa Fluor 488 in Ultra-Expansion microscopy. A max projection of an unprocessed Z-stack image with 75 images taken at a distance of 140 nm is shown. Two sections (*right*) are shown enlarged, to indicate the particles.

We attempted immunofluorescence detection of both fusion proteins with anti-BirA and anti-HA, as well as the detection of autobiotinylation of the respective baits using fluorescent streptavidin to confirm correct localization. Anti-BirA produced a weak signal, in particular for TEP1, albeit sufficient to reveal localization of the two baits to cytoplasmic particles (Fig. 3), with a pattern consistent with the eYFP signal from ^eYFP^MVP1. Detection with anti-HA and fluorescent streptavidin, both probes proven to faithfully detect TurboID-2xHA baits for various targets in different compartments (34, 35, 36), failed. This, together with the poor anti-BirA signal for TEP1, indicates antibody and streptavidin accessibility problems potentially caused by the vault shell. Indeed, when we applied expansion microscopy for TurboID-2xHA-TEP1 detection, we readily obtained a dot-like signal with fluorescent streptavidin, indicating correct localization to vault particles (Fig. 3*C*).

The cell lines were further analyzed by Western blotting using streptavidin to detect proximity-labeled proteins and anti-BirA to detect the bait (Fig. S6). Biotinylated proteins were readily detectable in all lines except for the parental control. For TurboID-2xHA-MVP1, bands consistent with the molecular weight of tagged and untagged MVP1 were dominant, with the latter also present in the TurboID-2xHA-TEP1 line. Next, whole cell lysates were subjected to streptavidin affinity purification followed by LC-MSMS analysis, as previously described (34). Proteins enriched, compared to parental controls, were grouped into confidence intervals (SigA, SigB; see Methods part), based on statistical analysis in Perseus (37) (Fig. 3, Table S2). An eYFP-TurboID-expressing cell line was used as an additional stringent control to define bystander labeling, which is expected to be considerable for cytoplasmic bait proteins (34).

The MVP1 and TEP1 TurboID interactomes exhibited robust agreement with each other, despite TEP1 being only mildly enriched with the MVP1 bait. The serine and arginine-rich (SR)-domain proteins TSR1 and RBSR1 were found enriched for both baits (Table S2). Consistently, a recent study found all three MVP isoforms immunoprecipitating with RBSR1 (Tb927.9.6870), together with a range of splicing factors, including TSR1 and U5-200k (38). RBSR2 (Tb927.7.1390), which was also pulling down MVPs in the same study (along with TSR1, RRM1, PRP19, U2 Ssm, and basal body components), was not detected in any of our approaches.

### The vault interactome is enriched in splicing factors

A high-confidence cohort of protein groups common between TurboID and affinity capture (enriched in both, TEP1 and MVP1 TurboID, and at least one affinity capture experiment) contained 103 protein groups (Table S2). Among these were RBSR1, the nucleolar protein NOP56 (Tb927.8.3750), the AAA-ATPase Tb927.11.13360 with potential nucleoplasmic localization (TrypTag; (28)), the universal minicircle sequence binding proteins 1 and 2 (UMSBP1 and UMSBP2), and several splicing factors, including TSR1, TSR1IP, U5-200k, and U2AF35.

The two UMSBP paralogs are both carrying CCHC-type zinc finger domains (39). A function in chromatin remodeling and gene expression regulation was recently assigned for UMSBP2 (40), which localizes to the telomeres. UMSBP1 was localized to the mitochondrion in an earlier report (41), but a nuclear signal (nuclear lumen and nucleolar periphery) was observed by TrypTag upon C-terminal eYFP tagging (28). A further chromatin remodeler within our high-confidence cohort is the sirtuin Sir2rp1 (Tb927.7.1690), which functions in DNA repair and telomeric gene silencing (42).

In *T. brucei,* vtRNA was reported to be required for the production of SL trans-spliced mRNA (13). Consistently, we find spliceosomal components, including the U2 auxiliary factor component U2AF35. U2AF65 is also significantly enriched in TEP1 and MVP1 TurboID, but not in the pulldown.

The vast majority of vault particles are cytoplasmic (Fig. 1*B*), but many interactors are mainly localized to other compartments, *i.e.,* the nucleus for the spliceosomal components, and thus only a small subset of each protein could engage in an interaction with the vault particle. Notably, several factors associated with *trans-*splicing (TSR1, TSR1IP, U5-200k, and U2AF35) (43, 44) are found enriched in the high-salt affinity capture, implying that these are contained inside the vault particle. It is therefore tempting to speculate that these proteins are entering the particle bound to RNA, which would be consistent with a role of the vault in sequestering vtRNPs and potentially other snRNPs. Moreover, interestingly, an additional role in controlling mRNA stability in trypanosomes has been established for several basal splicing factors, *i.e.*, for SF1 (absent from our interactomes), U2AF35, U2AF65 (44), and also TSR1 and TSR1IP (45, 46), suggesting a potential role of vtRNA in this process.

To the best of our knowledge, the sole clear overlap with mammalian MVP interactomes is the presence of TEP1. Another prototypic component of mammalian vault particles is the Poly(ADP-ribose) polymerase 4 (PARP4) vPARP (9), one out of 16 members of the PARP enzyme family in humans, which generates ADP-ribose protein modifications (47). The sole *T. brucei* Poly(ADP-ribose) polymerase (Tb927.5.3050) (48) is more closely related to human PARP1 (44% sequence identity; 91% coverage) than human vPARP (27% sequence identity; 60% coverage), and we did not detect *T. brucei* PARP in any TurboID experiment or the high-salt affinity capture.

A recent approach employing proximity labelling to derive the interactome of the human vault (27) detected chaperons, prominently including the 70 kDa heat-shock protein (Hsp70) and for which the authors provided evidence for interaction with the mature human vault particle. Consistently, *T. brucei* Hsp70 and an Hsp83 homolog are part of our high-confidence cohort.

Altogether, our interactome approach, reliant on two orthogonal methods, delivered a high-confidence cohort of MVP and TEP1 interactors dominated by spliceosomal proteins. For some of these splicing factors, RBSR1, TSR1, and U5-200k, a recent study involving immunoprecipitation with RBSR1 as bait (38) offers additional orthogonal validation.

While previous attempts to establish a TEP1 interaction network with components of the splicing machinery (13) failed, we devise here a list of MVP and TEP1 interactors. Notably, our TEP1 TurboID approach can detect TEP1 complexes enclosed within the dense vault particle that are likely inaccessible with many other techniques. Altogether, our data support the possibility of direct engagement of vtRNA in the process of *trans*-splicing or mRNA degradation and opens new avenues for interrogating the precise role of vtRNA, TEP1, and the vault particle in splicing and beyond. For the vault particle, the precise mode of interaction with the vtRNP remains to be investigated, to establish whether the vtRNP is terminally sequestered, potentially undergoing snRNP recycling within the particle, or if it is allowed to cycle in and out dynamically.

### vtRNA is highly abundant and primarily in the cytoplasm

The cytoplasmic localization of MVP1-3 is different from the reported nuclear localization of the detected splicing factors. Likewise, vtRNA has been exclusively detected in non-nucleolar nuclear speckles (13, 49). This is unexpected but could be explained by the failure of macromolecular probes to enter the dense proteinaceous shell of the vault particle.

To investigate, we embedded procyclic trypanosomes in LR-white and immobilized thin slices of the embedded cells on poly-lysine slides. We then performed single-molecule RNA FISH (smFISH) using the branched DNA technology (50) of the ViewRNA ISH Cell Assay Kit (Thermo Fisher Scientific) on these slices. The embedding in the solid LR-white matrix ensures that only surface-exposed RNA molecules are accessible to the probe, and this has two major advantages: (1) every RNA target of the cell is equally accessible to the probes, independent on the subcellular localization; in other words, the vault particles have been opened by the diamond knife; (2) the exclusive probing on the surface of the resin massively reduces the number of detectable RNA molecules (2D *versus* 3D detection) and thus allows quantification even of highly abundant RNA species, including total mRNAs (51).

**Figure 4:**
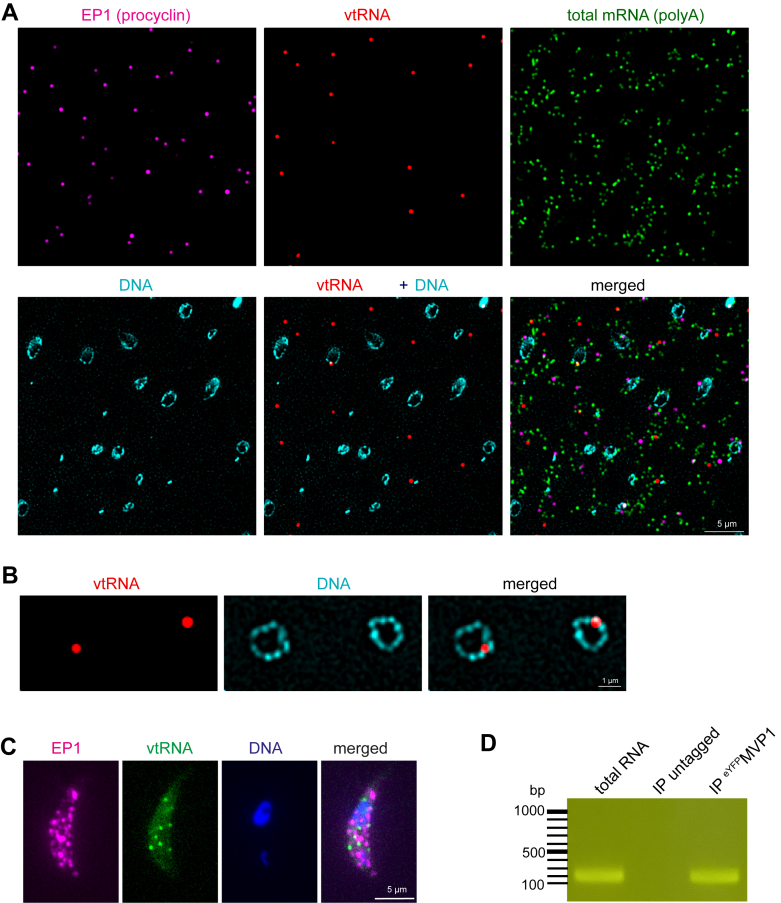
**Localization of vtRNA by smFISH on LR-White sections.** PCF trypanosomes were embedded in LR-white and single molecule FISH was performed on thin slices, immobilized on poly-Lysine slides. Images are shown as sum slices of a projection of 10 stacks (at 140 nm distances) and were processed by computational cleaning. The DNA was stained with DAPI and is shown in cyan. *A*, representative image (additional images are in Fig. S7). *B*, image showing vault localization to the nucleus. *C*, Localization of vtRNA by smFISH on whole cells. wt cells were probed for vtRNA and for EP1 mRNA. The number of detectable vtRNA molecules is low (<10 per cell). A representative image of a Z-stack projection (sum slices of 50 slices taken at 140 nm distances) is shown (additional images are in Fig. S9). *D*, vtRNA immunoprecipitates with ^eYFP^MVP1. Shown is an agarose gel analysis of products from RT-PCR with vtRNA specific primers of RNA eluted from the respective cryomilling affinity capture experiment. An IP with wt (untagged) cells served as control. For replicate experiments and Sanger sequencing results see (Fig. S10).

We probed for vtRNA, and, as controls, for EP1, one of the most abundant mRNAs in procyclic trypanosomes encoding the major cell surface protein, and for total mRNAs. DNA was stained with DAPI. We readily detected single dots for all three RNA species (Figs. 4 and S7), many dots for total mRNAs, and significantly less for the EP1 and vtRNA. As a control, we also probed for vtRNA together with negative controls against hygromycin and neomycin resistance gene transcripts that are not expressed in wild-type cells, and we got >100-fold fewer dots for the negative controls than for vtRNA, indicating that the vtRNA signal is specific (Fig. S8).

Quantifying the number of EP1 and vtRNA signals from six images, we found 3.2-fold fewer dots for vtRNA than for EP1 (n > 1200 vtRNA dots). However, EP1 was detected with 22 probe pairs, while the vtRNA probe consisted of only three probe pairs due to its smaller size. This means that the vtRNA abundance is underestimated. We have previously quantified the dependency between the number of probe pairs and the number of RNA dots on LR-white sections by probing the same RNA with a range of probe pairs (51): the difference in probe pairs between vtRNA and EP1 corresponds to a correction factor of three to 4. This means that the number of vtRNA molecules is roughly in the same range as the number of EP1 RNA molecules, which is about 500 molecules per cell (52).

We could readily detect vtRNA inside the nucleus (examples shown enlarged in Fig. 4*B*), consistent with the data of (13). However, the vast majority of vtRNA molecules (>90%) is unequivocally in the cytoplasm (Figs. 4*A* and S7), consistent with the localization of the vast majority of vault particles. The difference between the predominant cytoplasmic vtRNA localization when probed on LR-white slices and the exclusive nuclear localisation reported previously by standard FISH (13) suggests that the nuclear-localized vtRNA, or at least part of it, is vault particle-free.

### vtRNA co-immunoprecipitates with MVP1

As it remained unclear whether the cytoplasmic vtRNA is enclosed by the vault particle, we first attempted to co-stain the vault particles with vtRNA on the LR-white embedded cells. These attempts, deploying GFP-antibodies to detect N-terminal GFP-fusions of MVP1 and fluorescent streptavidin to detect autobiotinylation of N-terminal TurboID-2xHA of TEP1 or MVP1 fusions, failed to produce a robust, specific signal (data not shown). The likely reason is that the antigen concentration, restricted to the two dimensions of the LR-white section, is too low. Next, we attempted to stain vtRNA by smFISH in fixed whole cells. Interestingly, the number of detected vtRNA molecules, with <10 molecules per cell, was significantly lower than the number of EP1 mRNA molecules, which was, as expected, above the threshold that can be counted (Figs. 4*C* and S9). This is in stark contrast to the findings on LR-white embedded cells, where the numbers of vault and EP1 mRNA were roughly equal (see above). The likeliest explanation is that the majority of the vtRNA molecules are inside the vault particle, well protected from probe access, and only accessible when the particles are sliced open on the LR-white sections.

With imaging methods exhausted, we next attempted to demonstrate that vtRNA is associated with the vault particle biochemically. We carried out another cryomilling affinity capture experiment with the ^eYFP^MVP1 bait in the presence of RNAse inhibitors. Indeed, we could readily detect vtRNA in the proteinase K elution (Fig. 4*D*) by reverse transcription polymerase chain reaction (RT-PCR). Whilst this provided robust evidence that vtRNA is part of the vault particle, consistent with mammalian systems, it remained unclear if all cytoplasmic vtRNA is enclosed by a vault particle. However, the total number of vault particles, estimated from 2D images of the eYFP-fusion proteins, is in a similar range to the number of vtRNAs (Figs. 1*B* and 4). This would indicate that there are no or only few free vtRNAs, in particular, since the assumption is that every vault particle contains 12 vtRNAs (16). The absence of cytoplasmic free vtRNA is supported by the absence of a cytoplasmic FISH signal (13) and is in contrast to the findings in other systems, which report up to 95% of free vault RNA (16).

### MVP1 is dispensable for *T. brucei* viability under culture conditions

**Figure 5:**
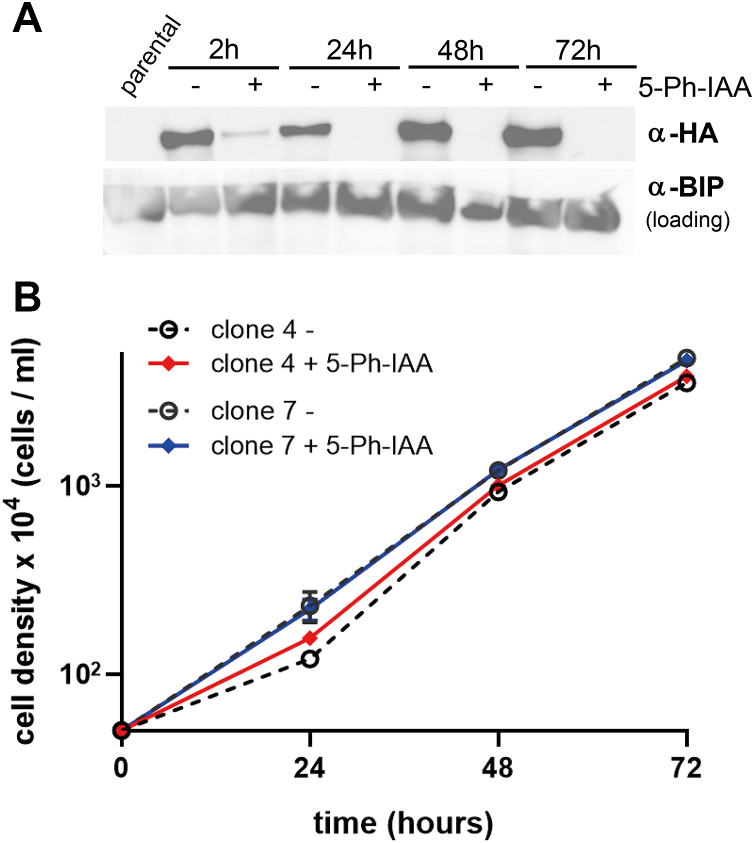
**Depletion of MVP1 does not impact *T. brucei* viability.***A*, MVP1 was depleted using a degron system based on induction with the auxin derivative 5-Ph-IAA (Fig. S11). Western blots for monitoring MVP1 depletion at 2 h, 24 h, 48 h and 72 h upon induction *via* the HA-epitope of the homozygous OsAID-3xHA-MVP1 fusion lines. Anti-BIP antibody stain served as loading control. *B*, for two clonal cell lines, growth was monitored in the presence and absence of 5-Ph-IAA. For the uncropped Western blot and biological replicates see Figure S12.

The apparent links to vtRNA and the splicing machinery prompted us to investigate the essentiality of the vault particle in *T. brucei.* To this end, we depleted MVP1 with an auxin-inducible degron system. Both alleles of the *mvp1* gene were N-terminally fused to the *Os*AID-3xHA sequence in a PCF line engineered for auxin-inducible degradation of the resulting fusion protein (35, 36) (Fig. S11). 2 h upon induction with the auxin analogue 5-Ph-IAA, MVP1 was decreased to approximately 7 (±2) %, then, from 24 h to 72 h, became undetectable by western blotting (Figs. 5*A* and S12). Monitoring growth over the same time-period revealed no difference to uninduced control (Fig. 5). For TEP1 we could not employ the degron approach, as the protein is likely protected from ubiquitination by the surrounding vault shell. Thus, we opted for inducible stem-loop RNAi that led to a modest decrease of TEP1 mRNA levels (to 77 ± 7%) (Fig. S13; Table S4) with the limited efficiency likely caused by the extreme sequence length (8418 bp). This *knockdown*, likewise, left *T. brucei* viability unaffected (Fig. S13). In conclusion, the trypanosome vault particle appears to be dispensable under normal culture conditions, consistent with the observed lack of phenotypic consequences upon *knockout* of mammalian MVP (18, 19) and TEP1 (12, 15).

### The vault shell is composed of all three MVP isoforms

**Figure 6:**
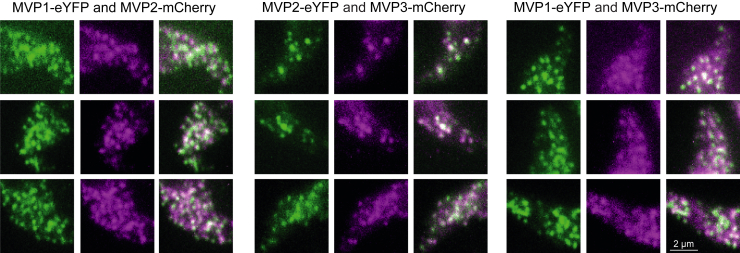
**The vault shell is composed of all three MVP paralogs.** Two of the three MVP paralogs (MVP1=Tb927.5.4460; MVP2=Tb927.10.1990; MVP3=Tb927.10.6310) were endogenously tagged in *T. brucei* PCF with eYFP and mCherry at the N-terminus in different combinations (as indicated above each *panel*) and subjected to fluorescence microscopy. Shown are single plane raw images for eYFP (*green*) and mCherry (*magenta*) of the anterior part of cells and a respective merge. The posterior part of the cell was excluded due to background signals arising from the lysosome and late endosomal compartments in the *red* channel (see Fig. S15).

The high enrichment observed for MVP2 and MVP3 throughout all MVP1 interactome analyses suggested that all three MVP paralogs are incorporated into a single vault shell. To test this hypothesis, we generated cell lines endogenously expressing all combinations of MVPs, with one fused to eYFP and the other to mCherry (Fig. S14). Monitoring fluorescence of the GFP variants showed co-localization of particles formed in all MVP combinations (Fig. 6). The mCherry signal intensity was lower, as expected, due to the lower quantum yield of this GFP variant. Monitoring the red channel produced a background signal at the posterior cell part, also present in *wt* cells used as control (Fig. S15), originating from a localization consistent with lysosomal and late endocytic compartments.

While the size of single vault particles of approximately 70 nm is below the resolution limit for light microscopy, the signal is amplified by the presence of multiple eYFP fusions per particle with its 78 MVP subunits. Hence, the observed colocalization data strongly suggest that each vault shell is a multimeric assembly of all three vault paralogs.

Consistently, Alphafold3 (53) modeling of heterotrimeric MVPs confidently predicts assemblies of MVP1, MVP2, and MP3, as well as respective homotrimers (Fig. S16).

Notably, evidence for vault particles composed of different isoforms has been described previously: *Dictyostelium discoideum*, phylum Amoebozoa, has two full-length MVP paralogs (Table S3). Disruption of either of these genes led to an altered vault morphology (54).

### Phylogenetic analysis of MVP paralogs

Phylogenetic reconstruction of MVP evolution based on animals, fungi, and plants concluded the gene was present in the last eukaryotic common ancestor (LECA) but lost in a number of groups, including fungi, insects, and plants (7).

**Figure 7:**
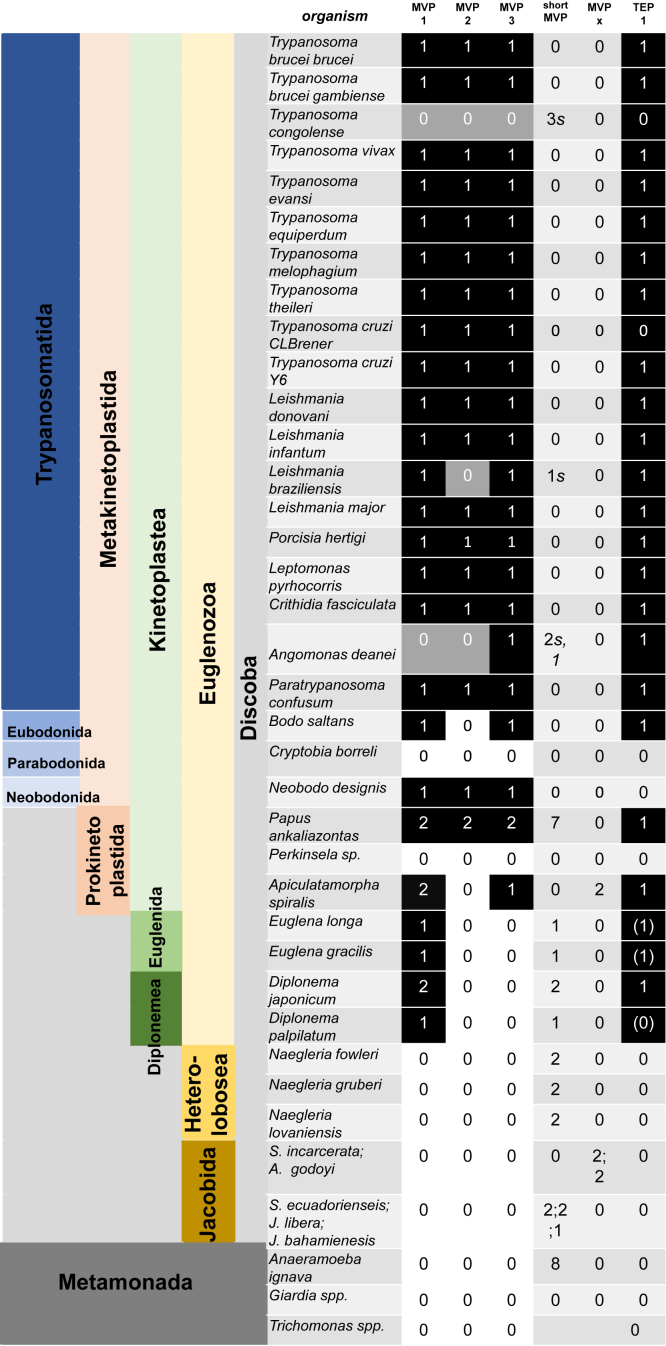
**Distribution of MVP paralogs and TEP1 within Discoba and Metamonada.** Numbers are given for the occurrence of TEP1 and MVP homologs, which were categorized by phylogenetic analysis into MVP1, MVP2, MVP3, and those which are not clustering (MVPx). *s* denotes synteny of short MVP isoforms with MVP1, MVP2 or MVP3 trypanosome genomic loci, suggestive of sequencing artefacts causing these partial absences. All sequences used are detailed and annotated in Table S3 and a corresponding phylogenetic tree is shown in Fig. S17.

With global vault phylogeny largely established, we set out to analyze the distribution of MVP in Discoba, the taxon to which *T. brucei* belongs, in more detail and with a focus on the occurrence of paralogs. Within the order of trypanosomatida, encompassing notorious pathogens as African and American trypanosomes and *Leishmania* spp., the vast majority of organisms encode three MVP paralogs that cluster within three separate clades (Table S3; Fig. S17). Absences or truncations may be attributed to genome quality as syntenic partial sequences could be detected. Within the phylum of Euglenozoa we detected the presence of MVPs except for *Perkinsela* sp., an obligate endosymbiont residing in *Paramoeba* (phylum Amoebozoa) which harbors a substantially reduced genome of ∼9.5 Mbp (55) and also the parabodonid *Cryptobia borreli*. All other sampled representatives have MVP paralogs (Fig. 7; Table S3; Fig. S17), with omnipresence of MVP1 but absences of paralogs clustering within MVP2 and, in particular, MVP3 clades, or both. Notably, other than previously assumed (7), this includes euglenids and diplonemids that contain two MVP paralogs in their transcriptome and genome sequences that became available recently (56, 57). One of those is clustering within the MVP1 clade and the second is not attracted to either the MVP2 or MVP3 clade. Jacobida and Heterolobosea, represented by *Naegleria* spp., lack full-length MVP genes altogether, but encode short MVP forms. Within the neighboring phylum Metamonada, MVP homologs are missing in prototypic organisms, including *Giardia* and *Trichomonas* species, but we detected eight MVP orthologs in the divergent metamonad *Anaeramoeba ignava* (58) which form a cluster close to the base of the MVP1 clade. Mammalian (*Rattus norvegicus*), as well as Amoebozoan (*D. discoideum, Mastigamoeba balamuthi*) MVPs, included due to their relevance in previous vault studies, are likewise neighboring the MVP1 clade (Fig. S17). Significantly, the two paralogs present in the Amoebozoa representatives, share high-sequence similarity which is in stark contrast to the divergence observed for the three Kinetoplastida MVP variants.

TEP1 occurrence appears to correlate with the presence of MVPs. It is omnipresent in Trypanosomatida, albeit with significant divergence of the C-terminal part of the large protein (2806 amino acids in *T. brucei*), downstream the WD40 domain (from *Tb*TEP1 position 2121), outside trypanosoma species. Despite the failure to detect full homolog sequences (Fig. 7; Table S3) in many other members of Euglenozoa, the sporadic presence across bodonids, Prokinetoplastida, and Diplonema, as well as partial sequences detected in euglenids, suggest TEP1 presence. In contrast, TEP1 appears to be absent in the remaining taxa (Jacobida, Heterolobosea) and in the phylum Metamonada.

Altogether, within Discoba, MVPs appear to be omnipresent in Euglenozoa, with the exception of the endosymbiont *Perkinsela.* In Jacobida and Heterolobosea only short MVP forms were detected. The occurrence of three significantly divergent paralogs appears to be a feature unique to Kinetoplastida.

## Conclusion

We show here that trypanosomes have vault particles and provide evidence that these are multimeric assemblies composed of three divergent MVP paralogs. Whilst the occurrence of MVP paralogs is not restricted to this taxon, and evidence for heteromultimeric MVP assemblies has been obtained in Amoebozoa (54), a tripartite MVP repertoire, featuring a considerable degree of divergence between paralogs, is remarkably conserved within Kinetoplastida.

Our comprehensive interactome approach delivers an inventory of high-confidence MVP interactors warranting further investigation to shed light on the elusive function of the vault. Furthermore, our data complement the recent discovery of the role of vtRNA and TEP1 in trypanosome mRNA splicing (13) and correct the localization pattern of trypanosome vtRNA, previously described to be exclusively nuclear.

Importantly, our interactomes prominently include a cohort of spliceosomal components associated with TEP1 and the vault particle, offering new leads to explore the vtRNP's precise function in mRNA-processing and whether this process is a unique adaptation coinciding with the divergent mRNA metabolism in Kinetoplastida.

## Experimental procedures

### Cell culture

PCF cells derived from *Trypanosoma brucei* subspecies *brucei* strain Lister 427 were grown in SDM-79 media (Life Technologies) supplemented with 25 mg/l of hemin (Sigma-Aldrich), 10% heat-inactivated fetal bovine serum (FBS) and 10 U/ml of penicillin/streptomycin (Thermo) in non-vented flasks at 27 °C. Respective bloodstream form (BSF) cells were cultivated at 37 °C in 5% CO2 in HMI-9 containing 10% (v/v) FBS. Cells were maintained at densities within their logarithmic growth phase.

### Genetic modifications

MVP coding sequences were endogenously tagged using the PCR-based pMOT (59) and pPOT7 system (60). pPOT7 was modified to result in fusing TurboID and a TurboID-2xHA-tag (34) or OsAID-3xHA (35, 36) to the N terminus of the protein. To generate tetracycline inducible RNAi cell lines in *T. brucei* BSF and PCF, we used the pRPaiSL (61) or the p3666 (62) based stem-loop RNAi system, respectively, using primers predicted by RNAit2 (63). RNAi lines were confirmed by quantitative RT-PCR (Table S4) using the Maxima H Minus cDNA Synthesis Master Mix (Thermo) and Maxima SYBR green qPCR master mix (Thermo) and a Biorad CFX96 device with 95 °C/10 min (initial denaturation), then 95 °C/15 s (denaturation), 60 °C/30 s (annealing), and 72 °C/40 s (extension) over 40 cycles. *Tb*GAPDH served as endogenous reference for normalization. All primers are listed in Table S1.

*MVP1 depletion by the auxin-inducible degron system*. Homozygous OsAID-3xHA-MVP1 fusion lines were confirmed by diagnostic PCR (Fig. S11). 50 μm 5-Ph-IAA (MedChem Express, HY-134653) was added to the cultured cells from a 50 mM stock in DMSO. The auxin system was kindly provided by Mark Carrington (University of Cambridge) and is described in (35, 36).

### Western blotting

Western blotting was performed using standard methods and horseradish peroxidase detection. Antibodies were anti-GFP (rabbit polyclonal; ab290, Abcam), anti-HA (rabbit polyclonal; PA1-985; Invitrogen or rat monoclonal; 3F10; Roche), anti-BirA (rabbit polyclonal; PA5-80250; Invitrogen) and anti-BIP (rabbit antiserum; Jeremy Bangs). Cy5-streptavidin was from Cytiva. All blots contained a parental control and were imaged on a ChemiDoc Imager (Biorad). *Os*AID-3xHA-MVP1 levels were quantified by densitometry using Fiji software. A corresponding BIP (Binding immunoglobulin protein) detection served for normalization.

### Flow cytometry

Clonal cell lines endogenously expressing MVP combinations, with one fused to eYFP and the other to mCherry were subjected to flow cytometry analyses on an LS Fortessa (BD Biosciences) using excitation wavelengths of 488 nm and 561 nm, in combination with a FITC filter (λ_emission_ = 530 nm, bandpass = 30 nm) and a PE-Texas Red filter (λ_emission_ = 610 nm, bandpass = 20 nm), respectively, and counting 10,000 cells. Data were processed using BD FACSDiva software v8.0.1 using a refined gate based on size *versus* granularity to exclude cell debris. Parental cells were subjected to the same procedure in parallel as negative control.

### Cryomilling and immunoprecipitation

Two liters of PCF *T. brucei* were grown in 2 L roller bottles and harvested at a density below 2 × 10^7^ cells/ml. The cell pellet was washed with PBS supplemented with protease inhibitors (mini cOmplete cocktail, Roche) and snap frozen in liquid nitrogen. The frozen pellet was ground into a fine powder using a Cryomill (Retsch) and stored at −80 °C. For LC-MSMS analyses, six smidgen spoons of powder were resuspended in 6 ml extraction buffer (20 mM HEPES pH 7.4, 100 mM NaCl, cOmplete EDTA-free protease inhibitor cocktail) supplemented with either 0.1% Triton-X100 (reduced to 0.02% for the washing steps), 0.5% Triton-X100 (reduced to 0.1% for the washing steps) or 0.1% BriJ36. A high salt buffer (supplemented with 500 mM NaCl) and containing 0.1% Triton-X100, was also tried. Extraction and affinity capture on magnetic anti-GFP nanobody beads (GFP-Trap Magnetic Agarose; Chromotek) was performed essentially as described (64). Captured proteins were eluted by on-bead tryptic digest and analyzed by LC–MS/MS on an Ultimate3000 nano rapid separation LC system (Dionex) coupled to an Orbitrap Fusion mass spectrometer (Thermo Fisher Scientific). Data were analyzed as detailed previously (25).

### Affinity purification of biotinylated proteins, mass spectrometry and analysis

Affinity purification of biotinylated proteins and tryptic digest and peptide preparation were performed as described (34), except that 1 mM biotin was added to the on-bead tryptic digests, to improve elution of biotinylated peptides. Eluted peptides were analyzed by LC-MSMS on an Ultimate3000 nano rapid separation LC system (Dionex) coupled to an Orbitrap Fusion mass spectrometer (Thermo Fisher Scientific). Spectra were processed using the intensity-based LFQ in MaxQuant version 2.1.3.0 (65, 66) searching the *T. brucei brucei* 927 annotated protein database (release 64) from TriTrypDB (67). Analysis was done using Perseus (37) essentially as described in (25). Briefly, known contaminants, reverse and hits only identified by site were filtered out. LFQ intensities were log2-transformed and missing values imputed from a normal distribution of intensities around the detection limit of the mass spectrometer. A Student’s *t* test was used to compare the LFQ intensity values between the duplicate samples of the bait with untagged control (WT parental cells) triplicate samples. The -log10 *p*-values were plotted *versus* the *t* test difference to generate multiple volcano plots (Hawaii plots). Potential interactors were classified according to their position in the Hawaii plot, applying cut-off curves for significant class A (SigA; FDR = 0.01, s0 = 0.1) and significant class B (SigB; FDR = 0.05, s0 = 0.1). The cut-off is based on the false discovery rate (FDR) and the artificial factor s0, which controls the relative importance of the *t* test *p*-value and difference between means (at s0 = 0 only the *p*-value matters, while at non-zero s0 the difference of means contributes).

### MVP1 RNA immunoprecipitation sequencing

*T. brucei* PCF cryo-powders (^eYFP^MVP1 expressing and parental cells) were generated as described above except that the cell pellets were supplemented with 200 Units Ribolock (Thermo) before cryomilling. All RIPseq buffers were prepared with diethylpyrocarbonate-treated, RNAse free water (Thermo). Eight smidgen spoons of powder were resuspended in 6 ml extraction buffer (25 mM HEPES pH 7.4, 100 mM NaCl, 0.1% BriJ36, 1 mM dithiothreitol (DTT), 10 Units/ml Ribolock. Extraction and pulldown were performed as described above using magnetic anti-GFP nanobody beads (Chromotek) blocked for 1 h with 0.15 ug/ml poly dI-dC (Roche) in extraction buffer prior to use (to restrict non-specific RNA binding). After four washing steps in washing buffer (25 mM HEPES pH 7.4, 100 mM NaCl, 0.02% BriJ36, 1 mM dithiothreitol (DTT), 10 Units/ml Ribolock), beads were subjected to proteinase K digestion in 100 ul PBS buffer containing 2% N-lauroylsarcosin, 10 mM ethylenediaminetetraacetic acid, 1 mM DTT, 100 units Ribolock and 20 ug Proteinase K (RNA grade; Thermo), incubating under shaking (900 rpm) at 42 °C for 1 h, followed by 55 °C for another hour. Beads were then removed, and the supernatants mixed with 350 ul buffer RLT (Qiagen) supplemented with 4 mM DTT. The eluted RNA was purified using the RNeasy Plus Mini kit (Qiagen) according to the manufacturer’s instructions, recovering in 20 ul RNAse free water. After pre-treatment with RNAse free DNAse I (Sigma), cDNA was prepared *via* random hexamers, using the Maxima H Minus cDNA Synthesis Master Mix (Thermo) according to the manufacturer’s instructions (25 °C for 10 min, 50 °C for 30 min and 85 °C for 5 min). The resulting cDNA was analyzed *via* PCR using vtRNA specific primers (Table S1). *T. brucei* whole cell cDNA served as positive control. The band containing the amplified product was extracted from the agarose gel, cloned into pJET1.2 (Thermo) and Sanger sequenced.

### Fluorescence microscopy, immunofluorescence and streptavidin detection of biotinylated proteins

For imaging direct eYFP and mCherry-fluorescence *Trypanosoma brucei* cells were harvested and resuspended in hemin- and FCS-free SDM79 medium, then fixed using 4% formaldehyde and directly applied to glass slides.

Images were acquired with a DMI8 widefield microscope (Thunder Imager, Leica Microsystems) with an HCX PL APO CS objective (100 x, NA = 1.4, Leica Microsystems). The microscope was controlled using LAS-X software (Leica Microsystems). Samples were illuminated with an LED8 light source (Leica). Images were captured using a K5 sCMOS camera (Leica, 6.5 μm pixel size). Images are presented as Z-stack projection (sum slices) of five slices a 140 nm distance for the smFISH experiments, and as single plane images for the eYFP and mCherry imaging.

Imaging of TurboID-HA tagged proteins was done with anti-HA (rabbit polyclonal; PA1-985; Invitrogen) or anti-BirA (rabbit polyclonal; PA5-80250; Invitrogen), in combination with anti-rabbit igG Alexa Fluor 488 (A-11008; Thermo Fisher Scientific) and Cy5-fluorophore labeled streptavidin (Cytiva), as described (68). For immunofluorescence, images were acquired by using Leica TCS SP8 WLL SMD-FLIM confocal microscope with a HC PL APO CS2 objective (63× Oil, NA 1.4, WD 0.14 mm, DIC) and Type F Immersion oil (NA:1.51). The microscope was performed with LAS-X software158 (Leica Microsystems). The excitation and emission light were separated by filter sets NF488 (excitation 495 nm–550 nm) for AlexaFluor 488 combined with SMD2 NF 405/640 (excitation 645–680) for Cy5-Streptavidin. Images were recorded by Z-stack between 30 and 40 images in 300 nm distances. Parental strains were used to control for unspecific signals. Images were processed without deconvolution in Fiji software.

### Expansion microscopy

Expansion microscopy was done using the UExM-protocol described in detail in (68).

*smFISH.* Single-molecule FISH on whole cells was done as described in (69). Briefly, we followed the Glass Slide format of the QuantiGene ViewRNA ISH Cell Assay Kit (Thermo Fisher Scientific), but we used a self-made washing buffer (0.1xSSC (saline sodium citrate); 0.1% SDS) for all but the last washing step in each of the washings to reduce costs. smFISH on LR-white embedded samples was carried out as described in (51). Briefly, cell pellets of PCF trypanosomes were high pressure frozen, freeze substituted and LR White-embedded as described (70, 71). 100 nm sections were cut with an ‘ultra Jumbo Diamond Knife’ (Diatome AG) and placed on a poly-lysine slide. Affymetrix smFISH was done on these slides essentially following the protocol of the QuantiGene ViewRNA ISH Cell Assay Kit, Glass Slide Format (Thermo Fisher Scientific), starting with the detergent step and omitting the protease step, and using the self-made washing buffer as above. The incubation with DAPI at the end was done for 30 min instead of 5, followed by three washing steps in PBS. For the detection of vtRNA we used three probe-pairs antisense to the entire length of the *T. brucei* vtRNA TBxRN-10. EP1 mRNA was detected by 22 probe pairs antisense to the ORF and both UTRs.

### Cisplatin dose-response analysis

Viability changes of *T. brucei* BSF in response to cisplatin treatment were assayed with fluorescence resazurin reduction assay, essentially as described (72). Briefly, 1.25 × 10^4^ cells/ml were exposed to a 19-concentration series of cisplatin dispensed using an ECHO650 acoustic dispenser (Beckman) in 384 well plates for 72 h, in triplicate, before resazurin addition. After further 14 h incubation, fluorescence intensity was recorded with a microplate reader (Synergy H1, BioTek) with λ_Ex_: 550 nm and λ_Em_: 590 nm. Percent of viability was calculated according to cell only and media controls.

### Bioinformatics analyses

MVP and TEP1 homologs in Discoba were detected by blast searching EuPathDB (73) and EUKPROT V3 (https://evocellbio.com/eukprot/). MVP alignments were generated in Clustal Omega (74) and edited using Jalview (75) retaining only unambiguous, homologous regions for phylogenetic analysis. Details of data sets are summarized in Table S3. Maximum-likelihood analysis was performed using PhyML (76) *via* the Phylogeny.fr pipeline (77) with 100 bootstrap replicates. Trees were edited in FigTree (http://tree.bio.ed.ac.uk/software/figtree/).

## Data availability

Proteomics data associated with these analyses have been deposited at the Pride Proteomics database at https://www.ebi.ac.uk/pridewithaccessionnumberPXD056004.

## Supporting information

This article contains supporting information.

## Conflict of interest

The authors declare that they have no conflicts of interest with the contents of this article.
