# Supplementary material for: The trypanosome vault particle is composed of multiple major vault protein paralogs and harbors vault RNA
Source: J Biol Chem. 2025 Sep 11;301(10):110706. doi: 10.1016/j.jbc.2025.110706 (PMC12547018; doi:10.1016/j.jbc.2025.110706)
Supplement: Supporting Figures and Tables [file mmc1.docx]

**Supporting Information for:**

**The trypanosome vault shell is composed of multiple major vault protein paralogs**

Anna Zavrelova^1*^, Siqi Shen^1*^, Farnaz Zahedifard^1^, Emmanuel Ayodeji Agbebi^1^, Silke Braune^2^, Susanne Kramer^2^ and Martin Zoltner^1
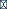
^

^1^ Department of Parasitology, Faculty of Science, Charles University in Prague, Biocev, Vestec, Czech Republic

^2^ Biocenter, University of Würzburg, Würzburg, Germany

**Supplementary Figures**

**Figure S1: MVP multiple sequence alignment.** The alignment shows MVP1 (Tb927.5.4460), MVP2 (Tb927.10.1990), MVP3 (Tb927.10.6310) and rat MVP (Q62667). Secondary structure elements, derived from the rat MVP X-ray structure (pdb:4V6O) are drawn below the alignment and respective domains (9 repeat domains (R1-R9), shoulder domain, cap-helix and cap-ring) are indicated.

**Figure S2: Western blot analysis of MVP1 endogenous N-terminal eYFP fusion**

The endogenous MVP1 eYFP fusion was detected by western blotting with an α-GFP antibody with a migration consistent with the theoretical molecular weight of (approximately 130 kDa (indicated).

**Figure S3: eYFP_**MVP1 assembles to particles localized in the cytoplasm. Shown are single plane raw images for eYFP (green) and DAPI (blue) fluorescence for >50 cells.

**Figure S4: Silver-stain SDS-PAGE analysis of ^eYFP^MVP1 pilot affinity capture and different extraction conditions.**

**Figure S5: MVP1 RNAi depletion does not affect cisplatin sensitivity.** Cisplatin sensitivity in *T. brucei* BSF was assessed by dose-response analysis via a resazurin based cell viability assay comparing the tetracyclin induced MVP1 RNAi line with uninduced control and the parental 2T1 strain.

**Figure S6: Western blot analyses of MVP1 and TEP1 endogenous TurboID-HA fusions.** Clonal cell lines from endogenous tagging were subjected to western blotting. (A) MVP1 fused to TurboID_HA was detected by an antibody against the HA epitope tag (right) at an apparent molecular weight consistent with the bait fusion construct (approximately 130 kDa, asterisk). Probing with Cy5-streptavidin (left) detects the same band and, additionally, a band migrating at the molecular weight of untagged MVP (approximately 95 kDa, double asterisk). Several other bands likely represent proximity labeled interactors. (B) While we failed to transfer the TEP1 fusion construct due to its high molecular weight (344 kDa), the Cy5-streptavidin decorated blot shows a band consistent with MVP (double asterisk). Note that the three MVP paralogs are indistinguishable in SDS- PAGE due to their almost identical molecular weight.

**Figure S7. Additional images for vtRNA localisation by smFISH on LR-White sections.** Procyclic trypanosomes were embedded in LR-white and single molecule FISH was performed on thin slices, immobilized on poly-Lysine slides. Images are shown as sum slices of a projection of 10 stacks (at 140 nm distances) and were processed by computational cleaning. Two representative images are shown with the DAPI stain DNA signal in cyan and the vtRNA smFISH signal in red.

**Figure S8. Negative controls for vtRNA smFISH.** The specificity of the vtRNA signal from smFISH on LR-White sections was validated by two negative controls, namely antisense probes to mRNAs encoding hygromycin and neomycin resistance proteins, respectively. Both genes are not expressed in wt cells, and consistently produced very low background signal.

**Figure S9: (A) Additional images for Figure 4. Localization of vtRNA by smFISH on paraformaldehyde fixed whole cells.** wt cells were probed for vtRNA and for EP1 mRNA. The number of detectable vtRNA molecules is low (<10 per cell). A representative image of Z-stack projections (sum slices of 50 slices taken at 140 nm distances) is shown. (B) vtRNA and EP1 smFISH on methanol fixed whole cells gives a similar signal ratio. Shown are single plane raw images of a Z-stack.

**Figure S10. vtRNA immunoprecipitates with ^eYFP^MVP1.** (A) Shown is an agarose gel analysis of products from RT-PCR with vtRNA specific primers of RNA eluted from the respective cryomilling affinity capture experiment (a replicate experiment for Figure 4D). An IP with wt (untagged) cells served as control. (B) Electropherogram from Sanger sequencing of the RT-PCR product. A sequence alignment with the coding sequence of *T. brucei* vtRNA is drawn on top.

**Figure S11.Confirmation *Os*AID-3xHA::MVP1 homozygous cell lines by diagnostic PCR.** (A) The auxin inducible degron system was employed for inducible degradation of *T. brucei* MVP1. Both endogenous alleles of the mvp1 gene were fused to *Os*AID-3xHA at the N-terminus. Two resistance cassettes were used, one with a puromycin and the other with a hygromycin resistance gene. The PCR strategy that was used to confirm the cell line and to control for the absence of the wild type allele, is schematically pictured. (B) PCR reactions were performed with a mixture of two forward oligos and products resolved on an agarose gel to discriminate wild type, heterozygous and homozygous cell lines. (C) 9 clones from the first transfection were positive (*), while only 3 clones out of 20 (10 shown) were homozygous for the *Os*AID-3HA fusion (**).

**Figure S12. Depletion of MVP1 using a degron system based on induction with the auxin derivative 5-Ph-IAA.** Western blots for monitoring MVP1 depletion in two clones at 2h, 24h, 48h and 72h upon induction via the HA-epitope. anti-BIP antibody stain served as loading control.

**Figure S13. TEP1 RNAi.** (A) TEP1 was depleted by inducible stem-loop RNAi which was confirmed by quantitative RT-PCR. A bar graph shows the level of TEP1 mRNA in the presence and absence of tetracyclin (Tet) for two clonal cell lines normalized using quantification of glyceraldehyde 3-phosphate dehydrogenase (GAPDH) mRNA (for raw data see Table S4). (B) Corresponding growth curve were recorded over a 72 h time period.

**Figure S14. Flow cytometry and Western blot analyses of double tagged cell lines.** Clonal cell lines endogenously expressing all combinations of MVPs, with one fused to eYFP and the other to mCherry were subjected to flow cytometry analyses. All three double-tagged lines show increased fluorescence at both wavelengths (A) in comparison to a wt control (B), proving expression of both GFP variants. These MVP fusions were detected by western blotting with an α-GFP antibody with a migration consistent with their molecular weight of (approximately 130 kDa; asterisk). Note that the three MVP paralogs are indistinguishable in SDS- PAGE due to their near identical molecular weight.

**Figure S15. The vault shell is composed of all three MVP paralogs.** MVP paralogs (MVP1=Tb927.5.4460 and MVP3=Tb927.10.6310) were endogenously tagged in *T. brucei* PCF with eYFP (MVP1) and mCherry (MVP3) at the N-terminus. Shown are single plane raw images for eYFP (green) and mCherry (magenta) and DAPI (blue) of a 1:1 mixture of eYFP_MVP1 cells and eYFP_MVP1/mCherry_MVP3 double tagged cells, and a respective merge. Autofluorescence background signals arising from the lysosome and late endosomal compartments in the red channel. However, as the endocytic system of *T. brucei is* entirely restricted to the part posterior of the nucleus, the anterior cell part remains unaffected by the background signal and thus can be used for colocalization -studies (see Figure 7).

**Figure S16: Alphafold3 models of trimeric MVP assemblies**

Trimeric MVP assemblies were modeled with Alphafold3 [50]. (A, B) The heterotrimeric *T. brucei* MVP model (TbMVP123; green cartoon depiction) exhibits high structural similarity with mammalian MVP (pdb:4V60; orange) as visualized by structural alignment. The position of the cap-helix relative to the repeat-domains, dependent on the bent in the shoulder domain, is different. (C) This is also the case when comparing an Alphafold model of trimeric rat MVP with the experimental structure (bottom). (D) Homotrimeric *T. brucei* MVP models (MVP111, MVP222, MVP333) adopt similar structures with variations of the shoulder domain bent.

**Figure S17: Phylogenetic tree of Discoba and Metamonada MVP sequences.** Selected organisms from Trypanosomatida (dark blue), Bodonids (light blue), Prokinetoplastida (orange). All sequences used are detailed and annotated in **Table S3**. Support values for nodes exhibiting >50% bootstrap support or better are indicated symbolically (see inset). Sequences of mammalian (*Rattus norvegicus)* and Amoebozoa (*Dictyostelium discoideum, Mastigamoeba balamuthi*) MVP (black) were included, as previous key studies were mainly performed in these phylae.

**­­Supplementary Tables**

**Table S1: Sequences of primers used in this work.** Sequences are written 5’ to 3’ and the purpose/plasmid system indicated for each used primer pair.

**Table S2: Mass spectrometry data.**

**Table S3: Species and genes used in phylogenetic analysis**

**Table S4: Source data for qRT-PCR analysis of TEP1 RNAi**
