# Supplementary material for: The trypanosome vault particle is composed of multiple major vault protein paralogs and harbors vault RNA
Source: J Biol Chem. 2025 Sep 11;301(10):110706. doi: 10.1016/j.jbc.2025.110706 (PMC12547018; doi:10.1016/j.jbc.2025.110706)
Supplement: Supporting Table S1 [file mmc2.docx]

| **Primers** | **sequence** | | | **purpose** |  |
| --- | --- | --- | --- | --- | --- |
| **Stem-loop RNAi** | | | | |  |
| Tb427.05.4460RNAi_F | GATCGGGCCCGGTACCTCTCTGCCAACAACCTCGTC | | | MVP1  RNAi |  |
| Tb427.05.4460RNAi_R | GATCTCTAGAGGATCCAAGCGTCTGTCTCCCAATGG | | | MVP1  RNAi |  |
| TEP1RNAi_SwaI_F | ATTTAAATTGTTAGGTCGTGGTCGGTTG | | | TEP1  RNAi |  |
| TEP1 RNAi_BamH1_R | GGATCCCGAATGCGCTGAAGAAGTCG | | | TEP1  RNAi |  |
| **Endogenous tagging with eYFP and *Os*AID_HA** | | | | |  |
| Tb927.10.6310_F | GGTGCGCGTAGATCATCTATTAAAGGAGGAAAAGAAAAAAAAATTGTTCACAGCAGCGTGAGCAGACAAGGTATAGGTGAGGTGTGATAgtataatgcagacctgctgc | | | YFP/Cherry tag |  |
| Tb927.10.6310_R | CGGTCCGGTGATAAGAACCGTACCATTATCGTTGTCGTTCGTGATGTGAGCATACTGATGAACCCCCAGTTTCAGGACCAGCTCATTCGCTAAATAATCATTCATACTACCCGATCCTGATCC | | | YFP/Cherry tag |  |
| Tb927.10.1990_F | GCAAATAAAAGTCAAGGATTGAAACCTTTTTTGTGTGATAGTATCGCTGTGTGTACGTGCCTAAGTATCTGAGGTCGGTAGGTAGTGAGAGgtataatgcagacctgctgc | | | YFP/Cherry tag |  |
| Tb927.10.1990_R | ACCTCACAAGTGTTCCTGTCCATTACATGAATATATTCATAAGGTCGCAACCTAATCACCTGATTCTCCTTGTCCACCATACTACCCGATCCTGATCC | | | YFP/Cherry tag |  |
| Tb927.5.4460_F | TATCTCTTAATATTTCAGCAATAACATCCAAGTTCAGAGGCGTT  GTCAATTTTCGTGTTGTCATTTTTGTCCAACTGTTTCGAAGAgtataatgcagacctgctgc | | | YFP/Cherry tag  and degron fusion |  |
| Tb927.5.4460_R | AGCATACATTTAGTGACATTTGTGTTGTTGTTCAGCAAATGAACGTAAAAATGACGTTTAATTCGTATGATATCACTCATACTACCCGATCCTGATCC | | | YFP/Cherry tag  and degron fusion |  |
| **Endogenous tagging with TurboID_2HA** | | | | |  |
| Vault_N_F primer | CTTAATATTTCAGCAGTAACATCCAAGTTCAGAGGCGTTGTCAATTTTCGTGTTGTCATTTTTGTCCAACTGTTTCGAAGGTATAATGCAGACCTGCTGC | | | MVP1  TurboID  tag |  |
| Vault_N_R primer | AGCATACATTTAGTGACATTTGTGTTGTTGTTCAGCAAATGAACGTAAAAATGACGTTTAATTCGTATGATATCACTCATACTACCCGATCCTGATCCAG | | | MVP1  TurboID  tag |  |
| TEP_N_F primer | GCGTGCAACGATGTGCGGTGAAGGCACGCGAGGTGCTTGCGGAGTACTCGCAGAACGTTTTGTCTCAAGTAGGTTTGCAGGGTTCTGGTAGTGGTTCC | | | TEP1 TurboID  tag |  |
| TEP_N_R primer | TTTCTCCTTGGAAAAAGATAACACCAGGCCCACCTTGCCCACGTTGGTGTTGCCTTCAGTTGGAAGACGTAACCACGCCACCAATTTGAGAGACCTGTGC | | | TEP1 TurboID  tag |  |
| **Confirmation of auxin degron *Os*AID** **fusions** | | | | |  |
| MVP1_AUX_5UTR_F | GCGACTTGAATCTGATGAGGTG | | | binds 605 bp upstream of the *mvp1* ATG, forwards |  |
| MVP1_AUX_ORF_R | GCTCCTTACGTGTGTACACCTG | | | binds 109 bp downstream of the *mvp1* ATG, reverse |  |
| OsAID_ORF_F | GAGCTGAGCATGGCCTTCCAG | | | binds within the *OsAID* fusion tag,  300 bp upstream of the *mvp1* ATG |  |
| **vtRNA primers for RIPseq/analytical PCR** | | | | |  |
| vtRNAF | GGGCGCAAGGATTTCGGATAC | | | Forward primer for RIPseq detection of vtRNA |  |
| vtRNAR | AGGGCGATTGCGGCTTCGGACAG | | | Reverse primer for RIPseq detection of vtRNA |  |
| **qPCR for validation of RNAi** | | | | |  |
| TEPqpcrF | | CACGCTGTTTCACACTGTGG | TEP1 qPCR | | |
| TEPqpcrR | | CCATGGTGTGCAGTTTGTTCC | TEP1 qPCR | | |
| TbGAPDHqPCR_F | | AGATTGATGTCGTTGCTGTTGTG | qPCR control | | |
| TbGAPDHqPCR_R | | ATGGCTTGCTCTTCGTAGTCG | qPCR control | | |
