# Supplementary material for: The trypanosome vault particle is composed of multiple major vault protein paralogs and harbors vault RNA
Source: J Biol Chem. 2025 Sep 11;301(10):110706. doi: 10.1016/j.jbc.2025.110706 (PMC12547018; doi:10.1016/j.jbc.2025.110706)
Supplement: Supporting Figure S1 [file mmc6.pdf]

Figure S1

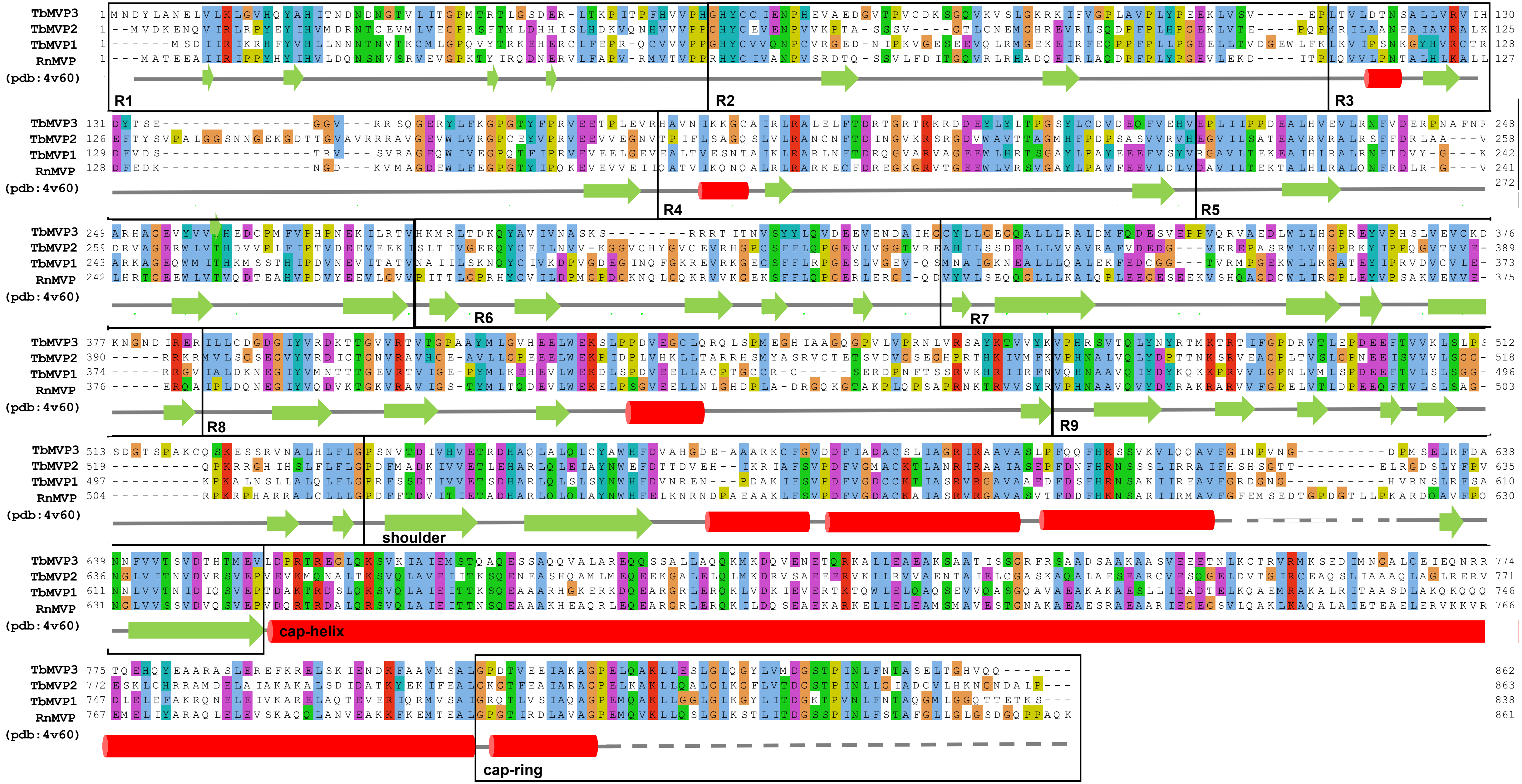

Figure S1: MVP multiple sequence alignment. The alignment shows MVP1 (Tb927.5.4460), MVP2 (Tb927.10.1990), MVP3 (Tb927.10.6310) and rat MVP (Q62667). Secondary structure elements, derived from the rat MVP X-ray structure (pdb:4V6O) are drawn below the alignment and respective domains (9 repeat domains (R1-R9), shoulder domain, cap-helix and cap-ring) are indicated.
