# Supplementary material for: The trypanosome vault particle is composed of multiple major vault protein paralogs and harbors vault RNA
Source: J Biol Chem. 2025 Sep 11;301(10):110706. doi: 10.1016/j.jbc.2025.110706 (PMC12547018; doi:10.1016/j.jbc.2025.110706)
Supplement: Supporting Figure S2 [file mmc7.pdf]

Figure S2

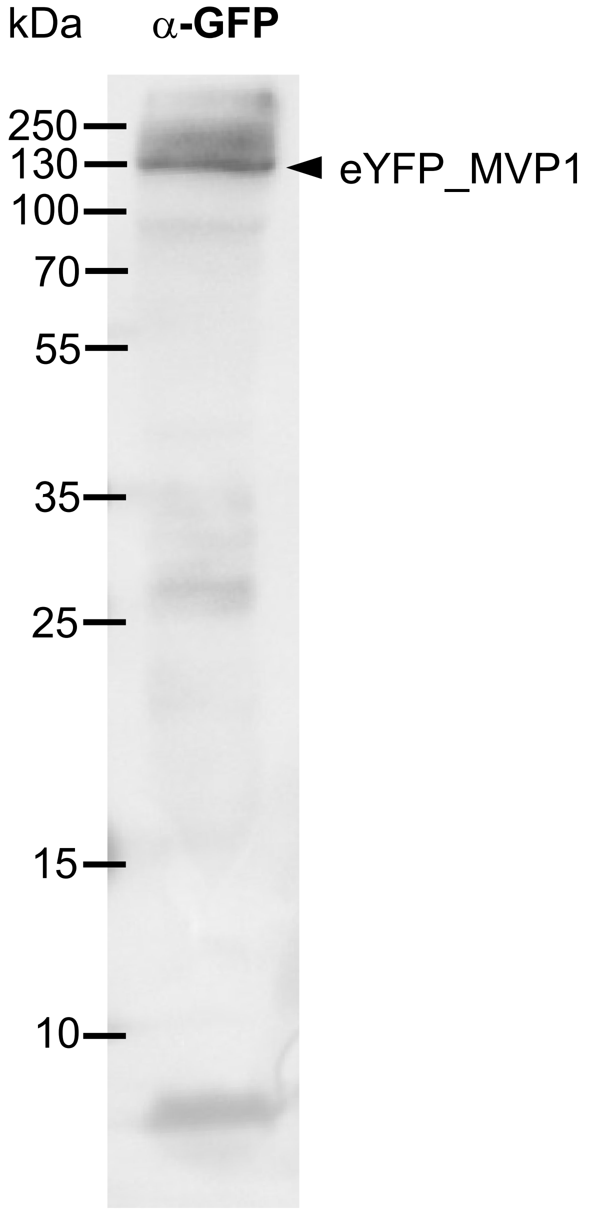

Figure S2: Western blot analysis of MVP1 endogenous N-terminal eYFP fusion. The endogenous MVP1 eYFP fusion was detected by western blotting with an α−GFP antibody with a migration consistent with the theoretical molecular weight of (approximately 130 kDa (indicated).
