# Supplementary material for: The trypanosome vault particle is composed of multiple major vault protein paralogs and harbors vault RNA
Source: J Biol Chem. 2025 Sep 11;301(10):110706. doi: 10.1016/j.jbc.2025.110706 (PMC12547018; doi:10.1016/j.jbc.2025.110706)
Supplement: Supporting Figure S3 [file mmc8.pdf]

# Figure S3

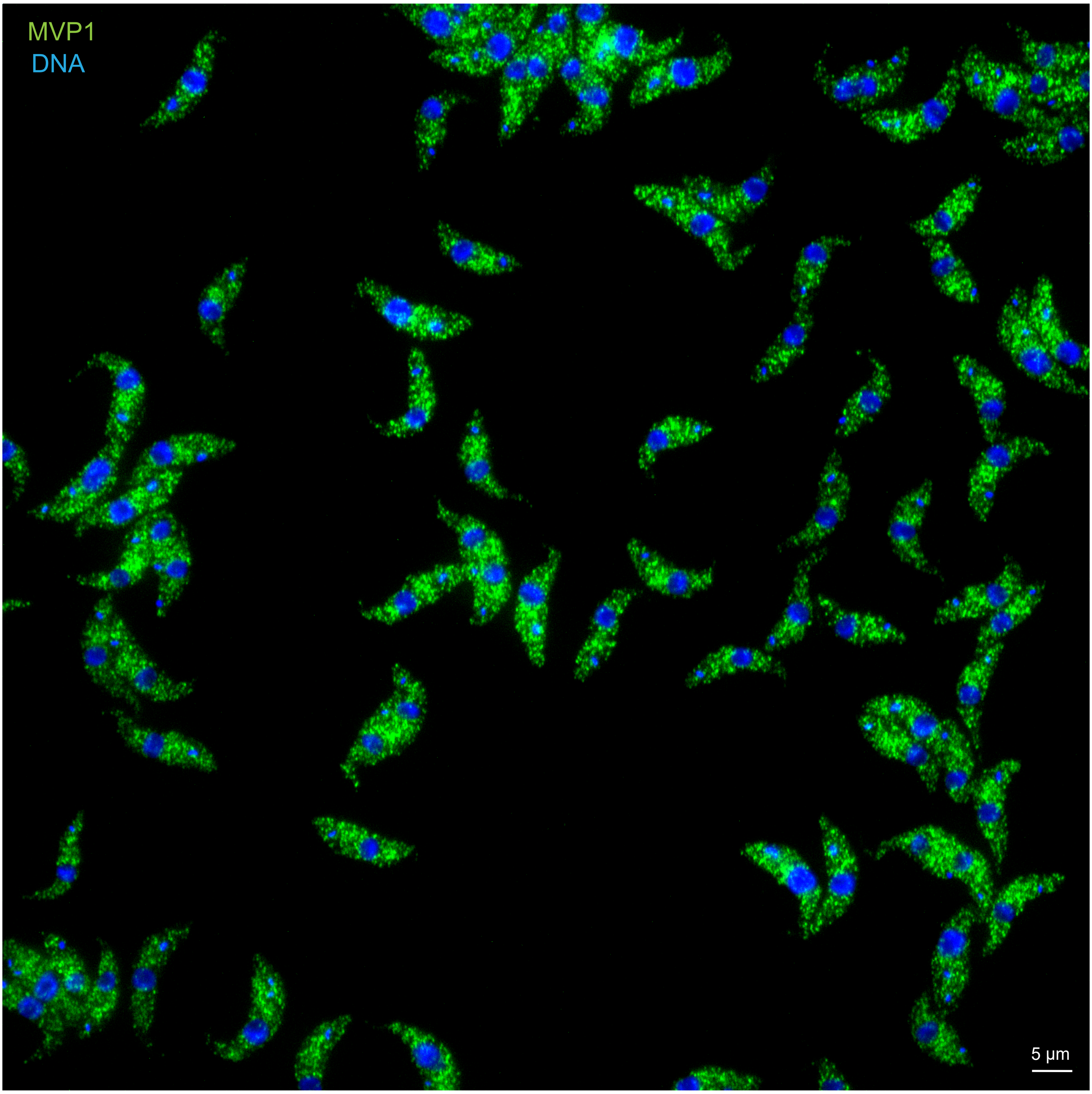


**Figure S3: eYFP_MVP1 assembles to particles localized in the cytoplasm.** Shown are single plane raw images for eYFP (green) and DAPI (blue) fluorescence for >50 cells.
