# Supplementary material for: The trypanosome vault particle is composed of multiple major vault protein paralogs and harbors vault RNA
Source: J Biol Chem. 2025 Sep 11;301(10):110706. doi: 10.1016/j.jbc.2025.110706 (PMC12547018; doi:10.1016/j.jbc.2025.110706)
Supplement: Supporting Figure S4 [file mmc9.pdf]

Figure S4

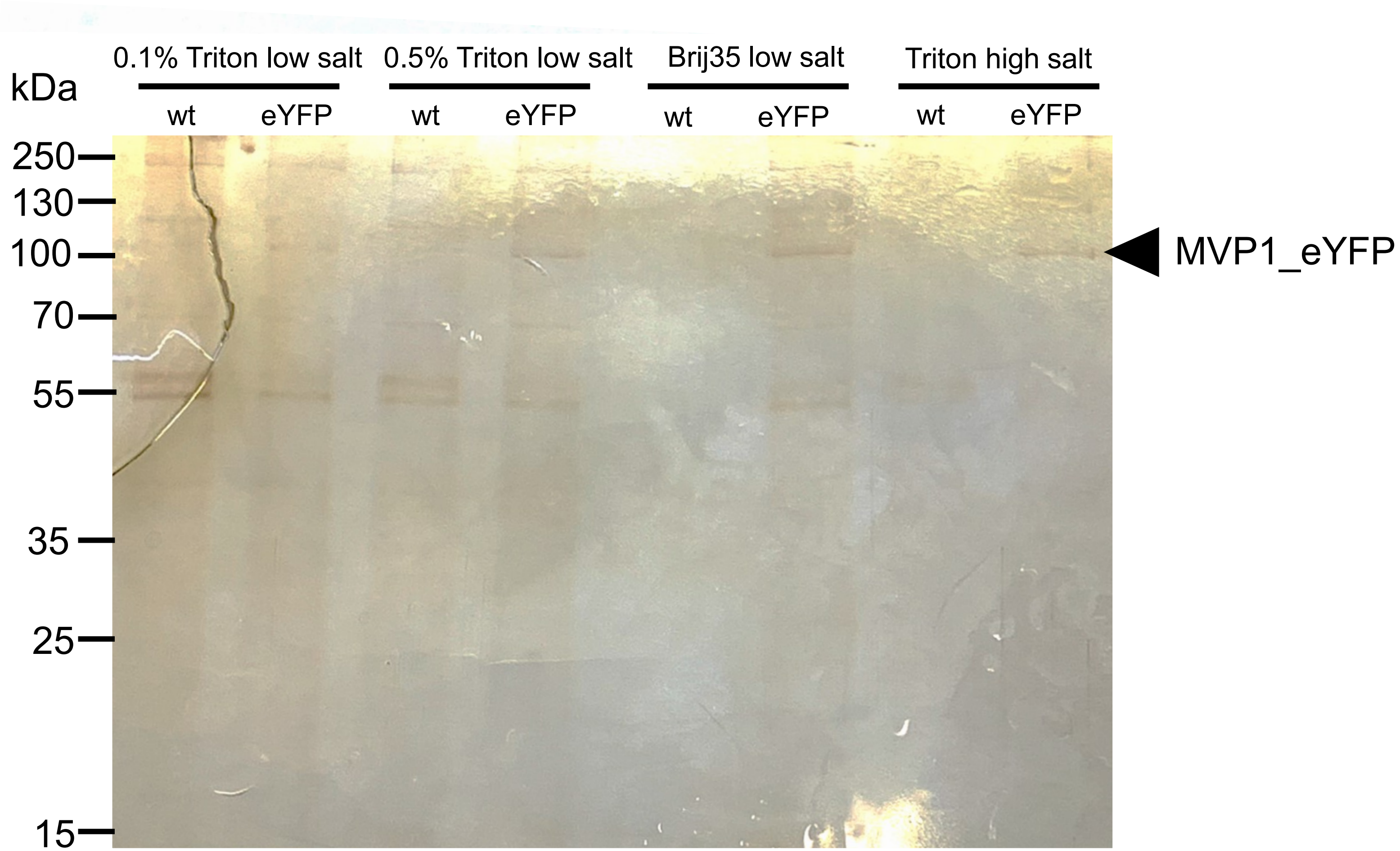

Figure S4: Silver-stain SDS-PAGE analysis of ^{eYFP}MVP1 pilot affinity capture and different extraction conditions.
