# Supplementary material for: The trypanosome vault particle is composed of multiple major vault protein paralogs and harbors vault RNA
Source: J Biol Chem. 2025 Sep 11;301(10):110706. doi: 10.1016/j.jbc.2025.110706 (PMC12547018; doi:10.1016/j.jbc.2025.110706)
Supplement: Supporting Figure S5 [file mmc10.pdf]

Figure S5

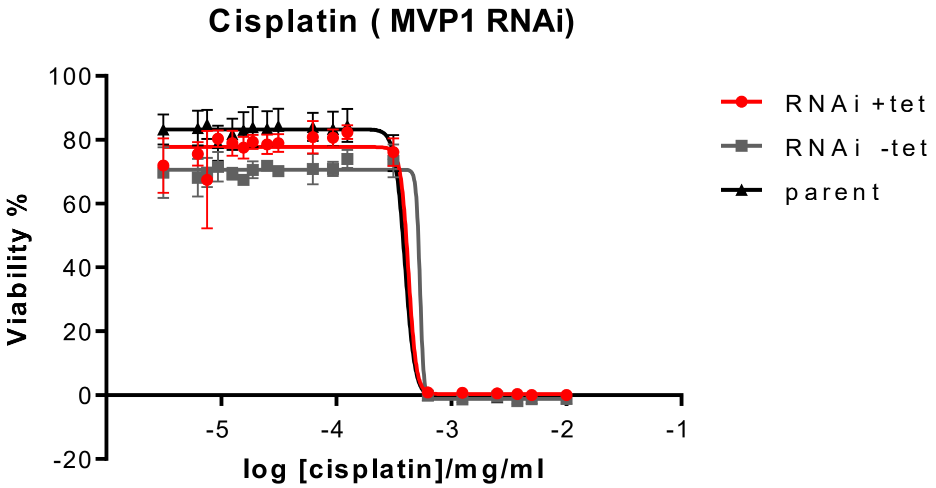

Figure S5: MVP1 RNAi depletion does not affect cisplatin sensitivity. Cisplatin sensitivity in T.brucei BSF was assessed by dose-response analysis via a resazurin based cell viability assay comparing the tetracyclin induced MVP1 RNAi line with an uninduced control and the parental 2T1 strain.
