# Supplementary material for: The trypanosome vault particle is composed of multiple major vault protein paralogs and harbors vault RNA
Source: J Biol Chem. 2025 Sep 11;301(10):110706. doi: 10.1016/j.jbc.2025.110706 (PMC12547018; doi:10.1016/j.jbc.2025.110706)
Supplement: Supporting Figure S6 [file mmc11.pdf]

Figure S6

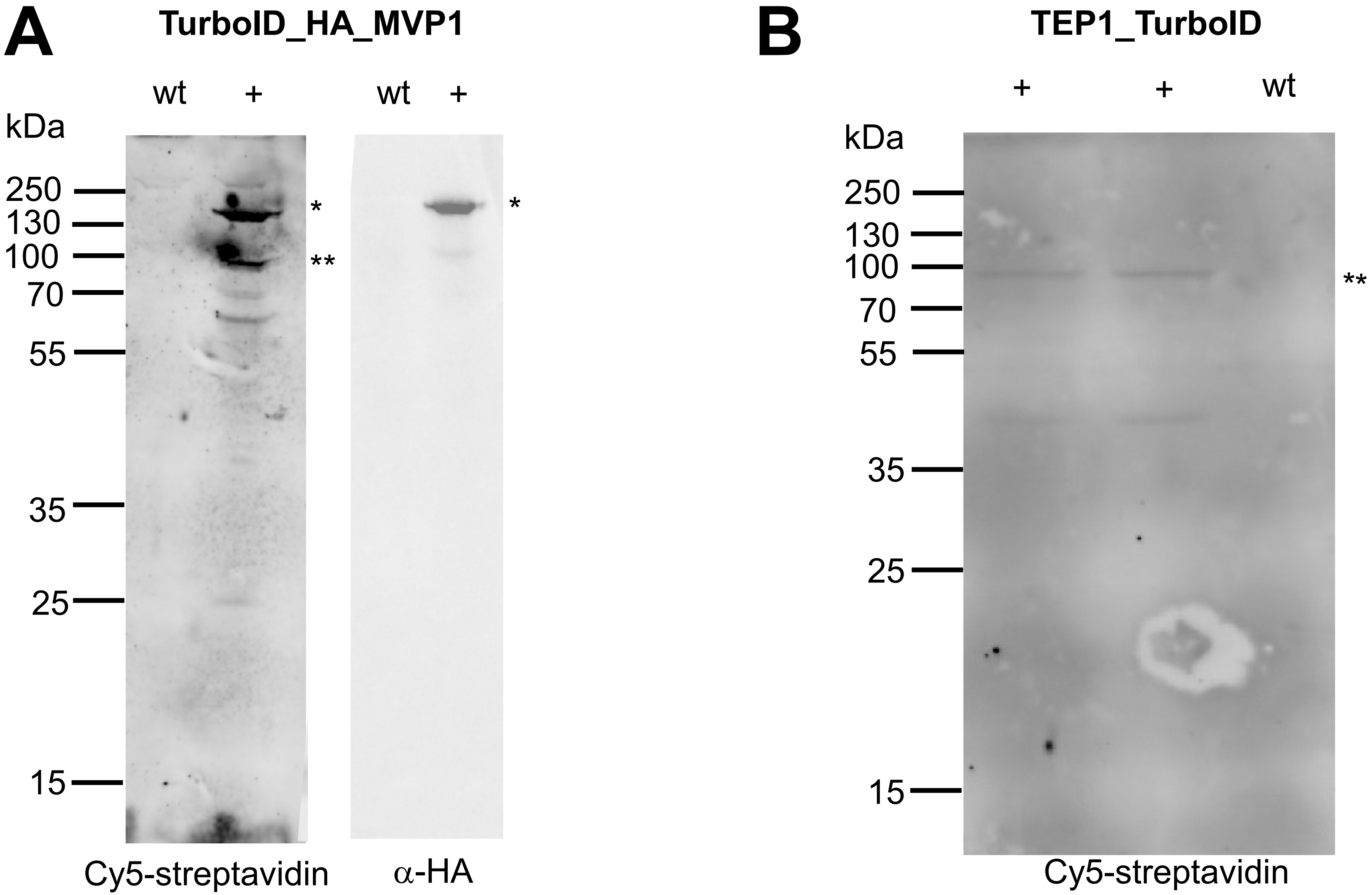

Figure S6: Western blot analyses of MVP1 and TEP1 endogenous TurboID-HA fusions. Clonal cell lines from endogenous tagging were subjected to western blotting. (A) MVP1 fused to TurboID_HA was detected by an antibody against the HA epitope tag (right) at an apparent molecular weight consistent with the bait fusion construct (approximately 130 kDa, asterisk). Probing with Cy5-streptavidin (left) detects the same band and, additionally, a band migrating at the molecular weight of untagged MVP (approximately 95 kDa, double asterisk). Several other bands likely represent proximity labeled interactors. (B) While we failed to transfer the TEP1 fusion construct due to its high molecular weight (344 kDa), the Cy5-streptavidin decorated blot shows a band consistent with MVP. Note that the three MVP paralogs are indistinguishable in SDS- PAGE due to their almost identical molecular weight.
