# Supplementary material for: The trypanosome vault particle is composed of multiple major vault protein paralogs and harbors vault RNA
Source: J Biol Chem. 2025 Sep 11;301(10):110706. doi: 10.1016/j.jbc.2025.110706 (PMC12547018; doi:10.1016/j.jbc.2025.110706)
Supplement: Supporting Figure S7 [file mmc12.pdf]

## Figure S7

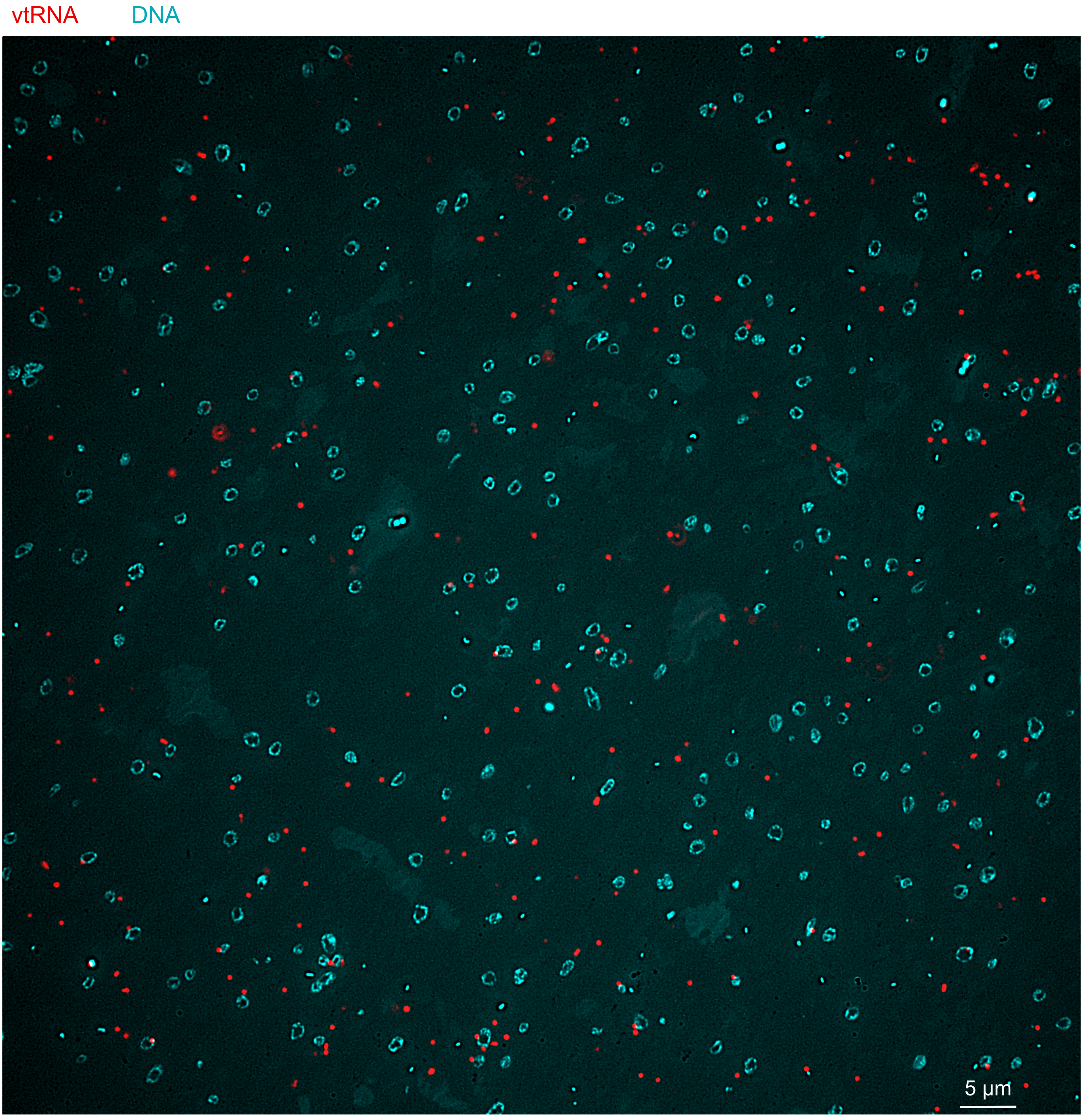


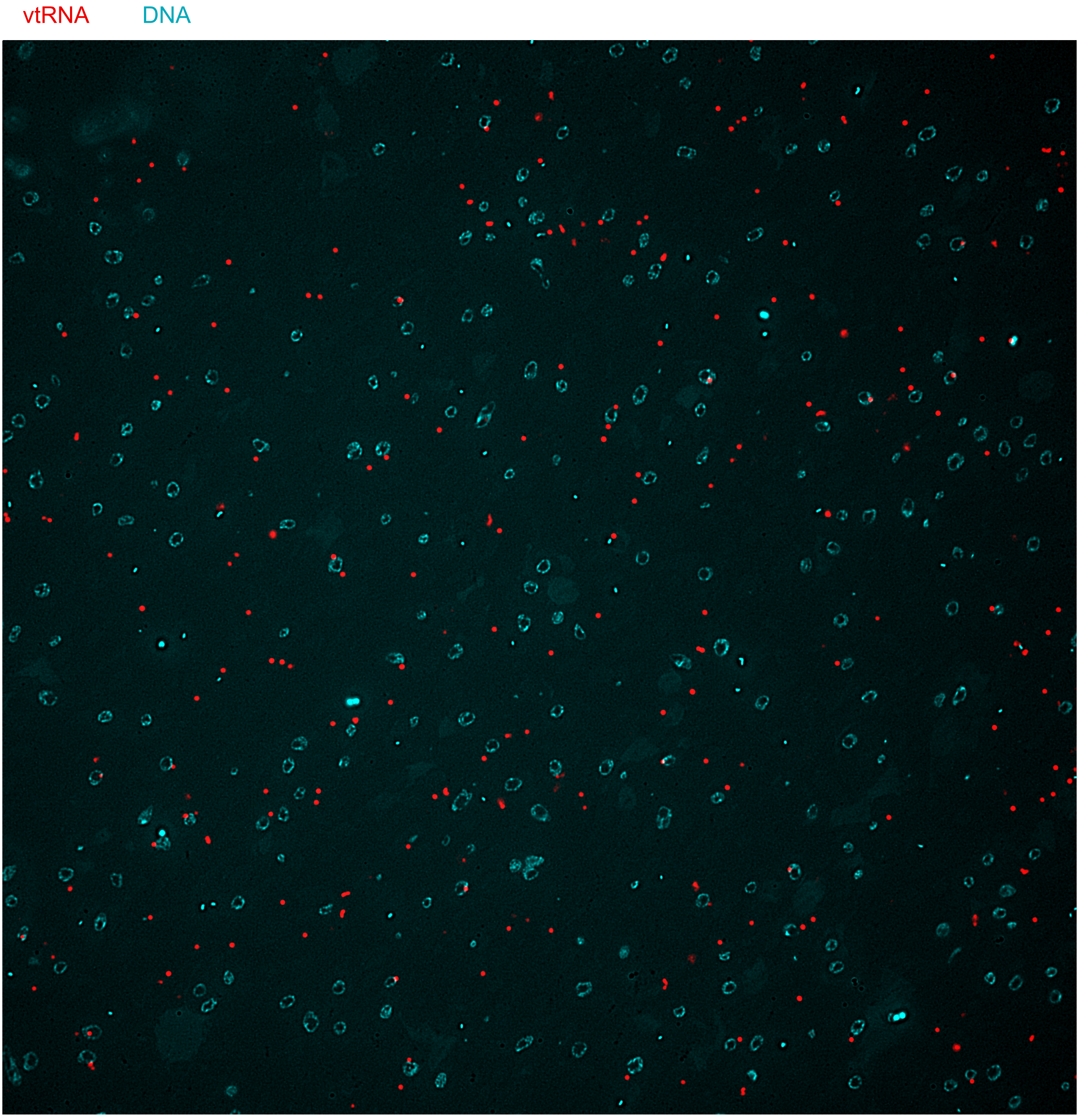


**Figure S7. Additional images for vtRNA localisation by smFISH on LR-White sections.** Procyclic trypanosomes were embedded in LR-white and single molecule FISH was performed on thin slices, immobilized on poly-Lysine slides. Images are shown as sum slices of a projection of 10 stacks (at 140 nm distances) and were processed by computational cleaning. Two representative images are shown with the DAPI stain DNA signal in cyan and the vtRNA smFISH signal in red.
