# Supplementary material for: The trypanosome vault particle is composed of multiple major vault protein paralogs and harbors vault RNA
Source: J Biol Chem. 2025 Sep 11;301(10):110706. doi: 10.1016/j.jbc.2025.110706 (PMC12547018; doi:10.1016/j.jbc.2025.110706)
Supplement: Supporting Figure S8 [file mmc13.pdf]

Figure S8

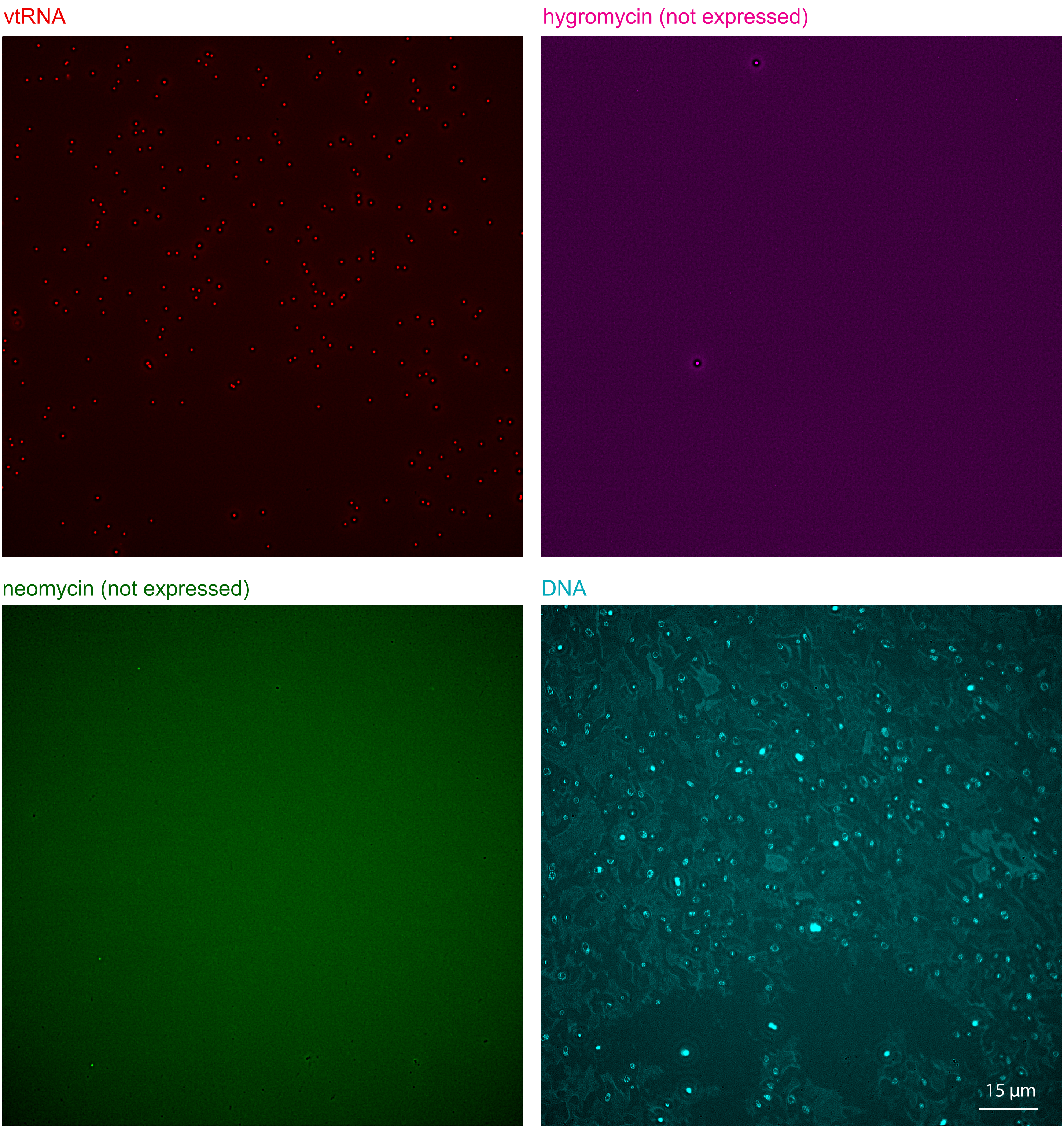

Figure S8. Negative controls for vtRNA smFISH. The specificity of the vtRNA signal from smFISH on LR-White sections was validated by two negative controls, namely antisense probes to mRNAs encoding hygromycin and neomycin resistance proteins, respectively. Both genes are not expressed in wt cells, and consistently produced very low background signal.
