# Supplementary material for: The trypanosome vault particle is composed of multiple major vault protein paralogs and harbors vault RNA
Source: J Biol Chem. 2025 Sep 11;301(10):110706. doi: 10.1016/j.jbc.2025.110706 (PMC12547018; doi:10.1016/j.jbc.2025.110706)
Supplement: Supporting Figure S9 [file mmc14.pdf]

Figure S9

A

EP1 vault RNA DNA merged

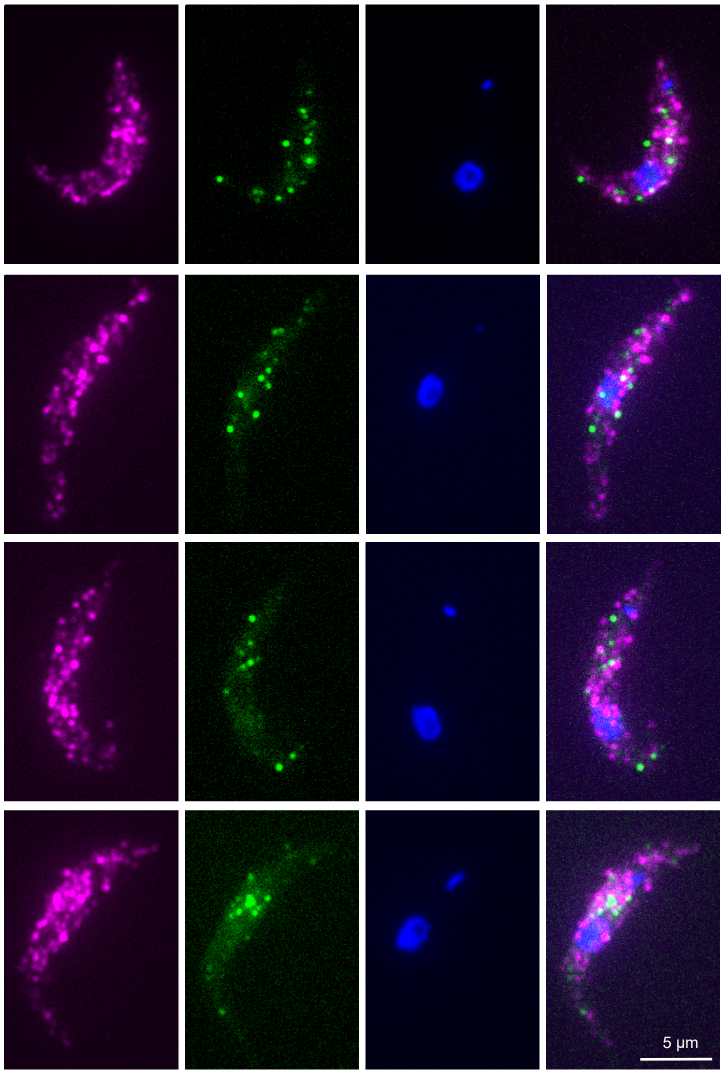

B

DIC + vtRNA vtRNA EP1

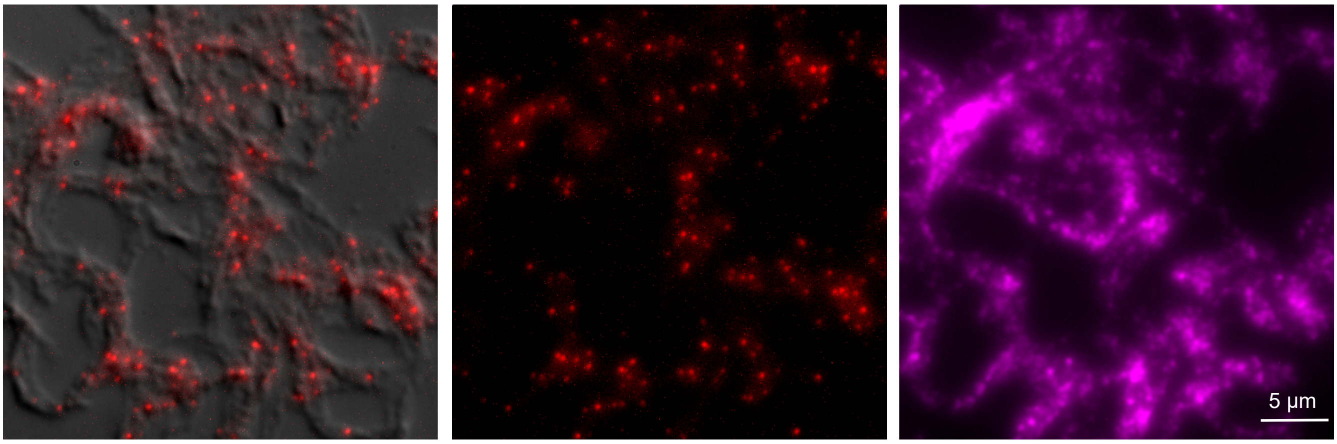

Figure S9: (A) Additional images for Figure 4. Localisation of vtRNA by smFISH on paraformaldehyde fixed whole cells. wt cells were probed for vtRNA and for EP1 mRNA. The number of detectable vtRNA molecules is low (<10 per cell). A representative image of Z-stack projections (sum slices of 50 slices taken at140 nm distances) is shown.**(B) vtRNA and EP1 smFISH on methanol fixed whole cells** gives a similar signal ratio.Shown are single plane raw images of a Z-stack.
