# Supplementary material for: The trypanosome vault particle is composed of multiple major vault protein paralogs and harbors vault RNA
Source: J Biol Chem. 2025 Sep 11;301(10):110706. doi: 10.1016/j.jbc.2025.110706 (PMC12547018; doi:10.1016/j.jbc.2025.110706)
Supplement: Supporting Figure S10 [file mmc15.pdf]

Figure S10

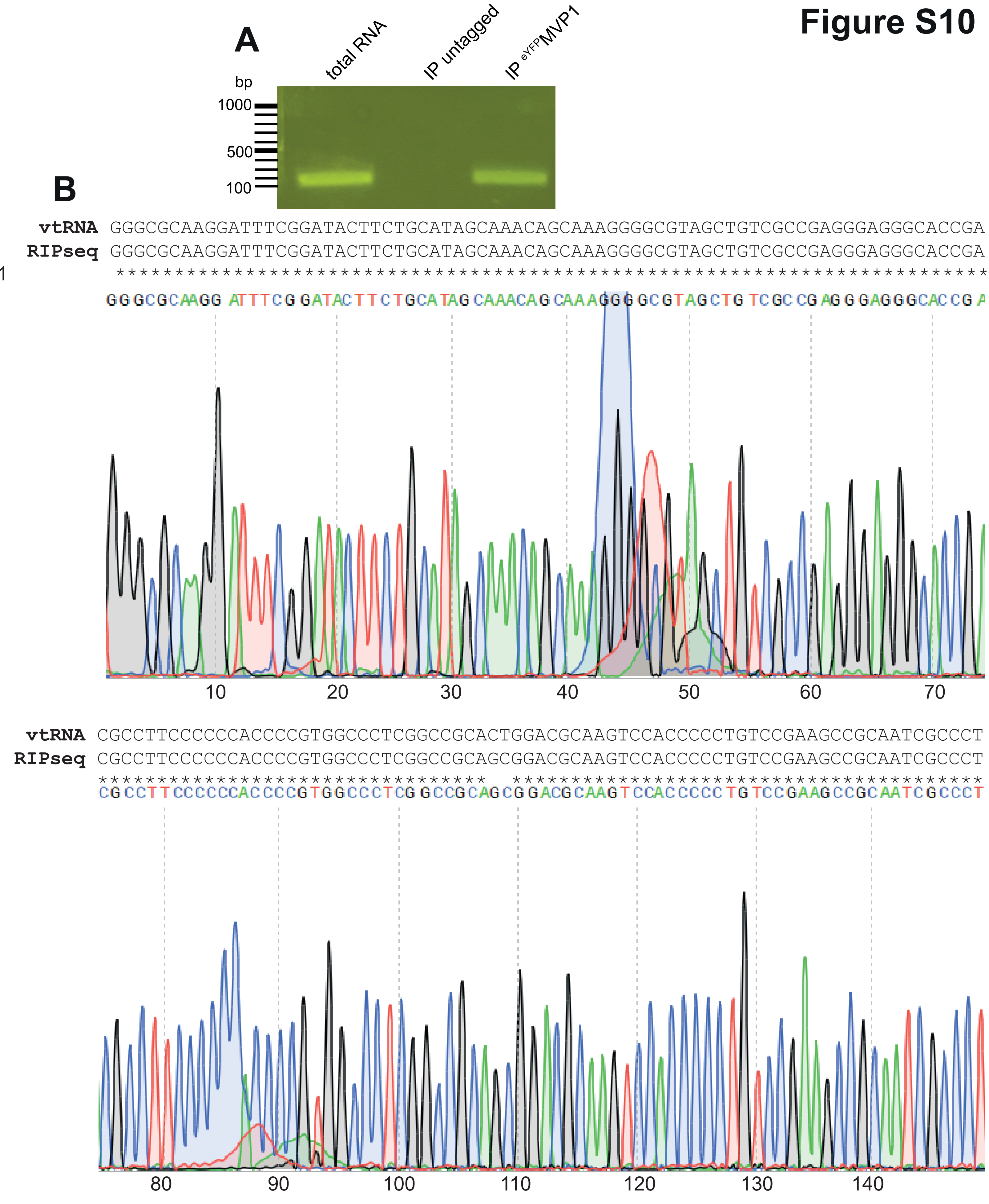

Figure S10. vtRNA immuno-precipitates wtth ^{eYFP}MVP1. (A)Shown is a replicate expeiment for Figure 4D, an agarose gel analysis of products from RT-PCR with vtRNA specific primers of RNA eluted from the respective cryomilling affinity capture experiment. An IP with wt (untagged) cells served as control.
(B) Electropherogram from Sanger sequencing of the RT-PCR product. A sequence alignment with the coding sequence of *T. brucei* vtRNA is drawn on top.
