# Supplementary material for: The trypanosome vault particle is composed of multiple major vault protein paralogs and harbors vault RNA
Source: J Biol Chem. 2025 Sep 11;301(10):110706. doi: 10.1016/j.jbc.2025.110706 (PMC12547018; doi:10.1016/j.jbc.2025.110706)
Supplement: Supporting Figure S11 [file mmc16.pdf]

Figure S11

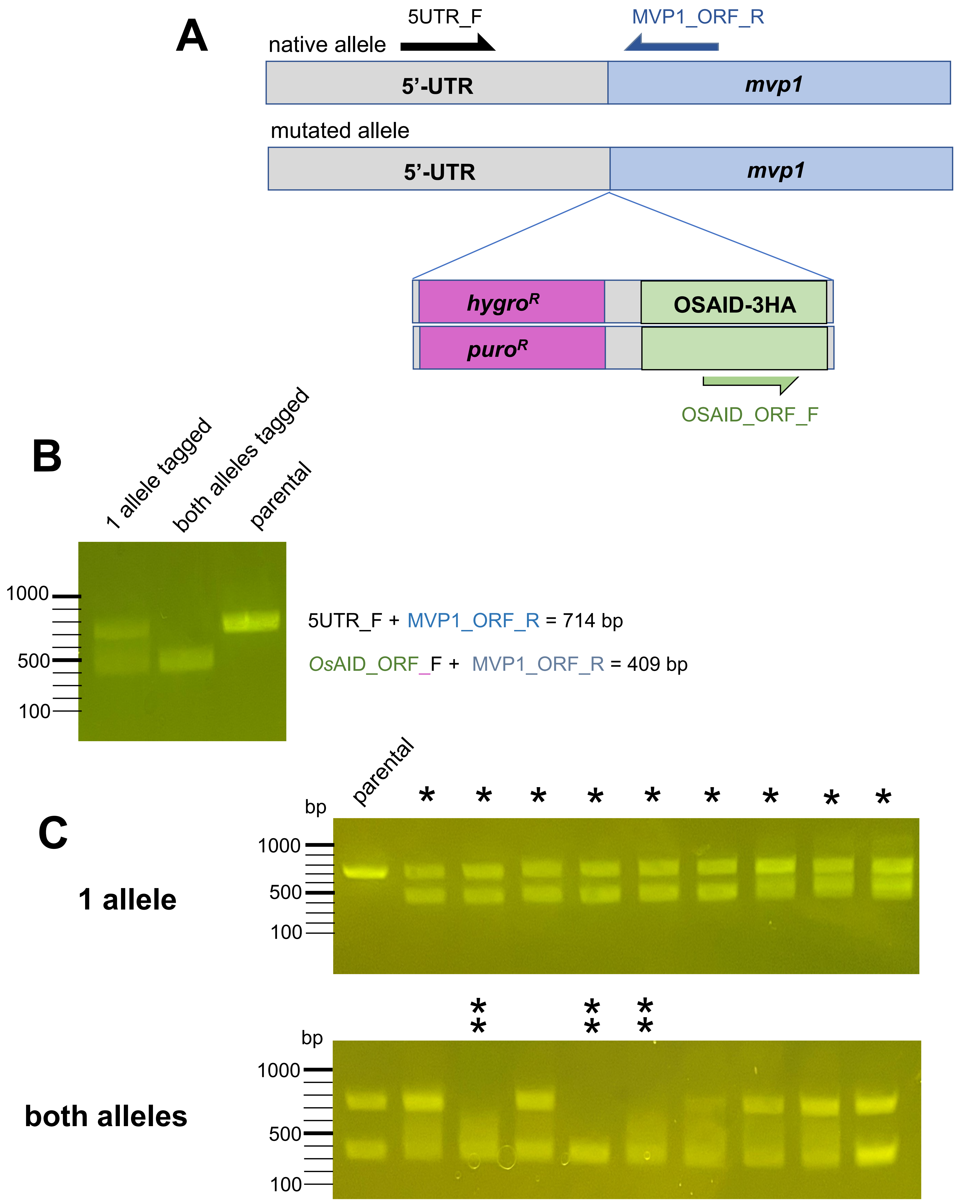

Figure S11.Confirmation OsAID-3xHA::MVP1 homozygous cell lines by diagnostic PCR.(A) The auxin inducible degron system was employed for inducible degradation of *T. brucei* MVP1. Both endogenous alleles of the mvp1 gene were fused to OsAID-3xHA at the N-terminus. Two resistance cassettes were used, one with a puromycin and the other with a hygromycin resistance gene. The PCR strategy that was used to confirm the cell line and, in particular, to control for the absence of the wild type allele, is schematically pictured. (B) PCR reactions were performed with a mixture of two forward oligos and products resolved on an agarose gel to discriminate wild type, heterozygous and homozygous cell lines. (C) 9 clones from the first transfection were positive (*), while only 3 clones out of 20 (10 shown) were homozygous for the OsAID-3HA fusion (**).
