# Supplementary material for: The trypanosome vault particle is composed of multiple major vault protein paralogs and harbors vault RNA
Source: J Biol Chem. 2025 Sep 11;301(10):110706. doi: 10.1016/j.jbc.2025.110706 (PMC12547018; doi:10.1016/j.jbc.2025.110706)
Supplement: Supporting Figure S12 [file mmc17.pdf]

Figure S12

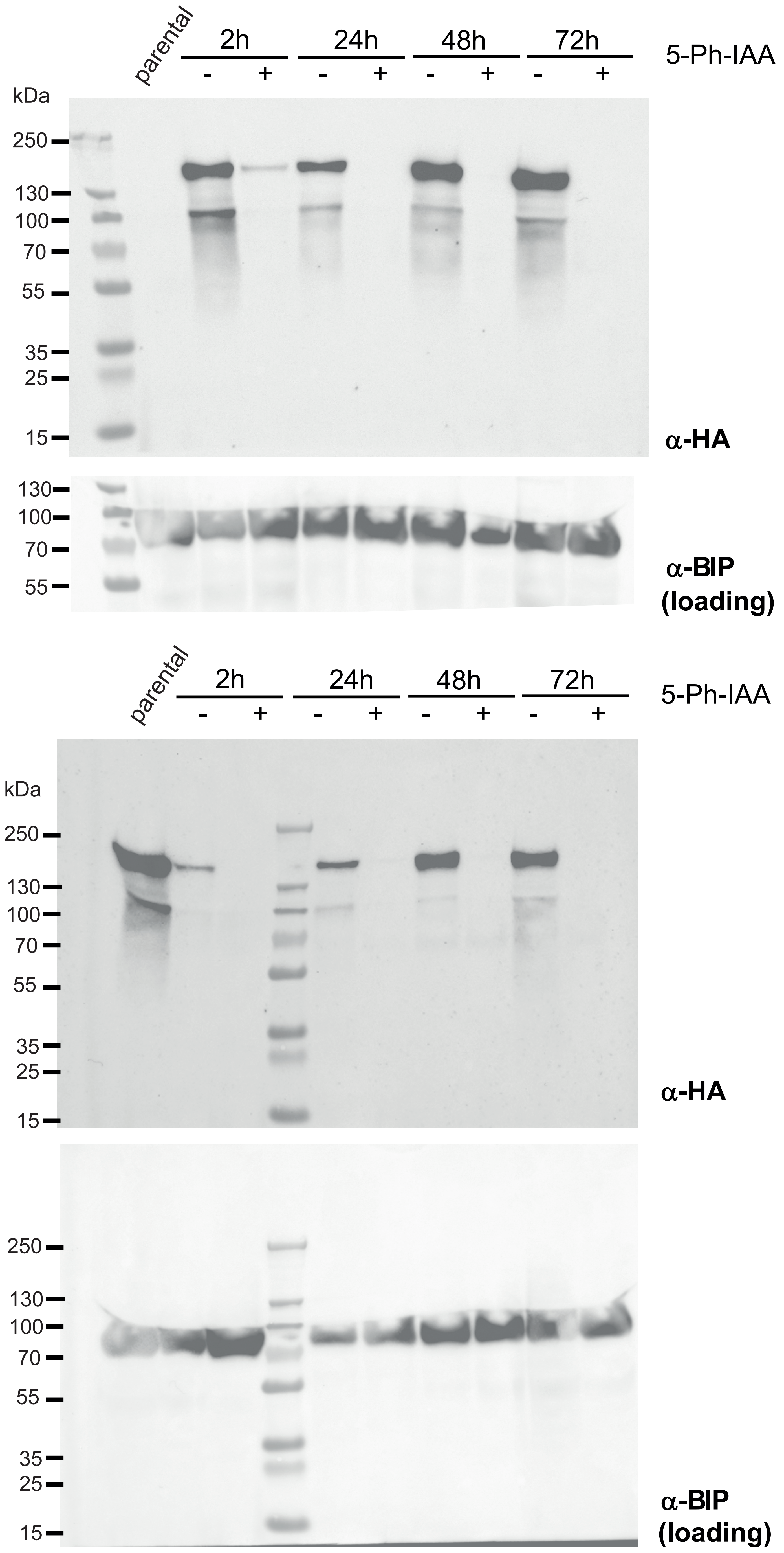

Figure S12. Depletion of MVP1 using a degron system based on induction with the auxin derivative 5-Ph-IAA. (A) Western blots for monitoring MVP1 depletion at 2h, 24h, 48h and 72h upon induction via the HA-epitope. anti-BIP antibody stain served as loading control.
