# Supplementary material for: The trypanosome vault particle is composed of multiple major vault protein paralogs and harbors vault RNA
Source: J Biol Chem. 2025 Sep 11;301(10):110706. doi: 10.1016/j.jbc.2025.110706 (PMC12547018; doi:10.1016/j.jbc.2025.110706)
Supplement: Supporting Figure S13 [file mmc18.pdf]

Figure S13

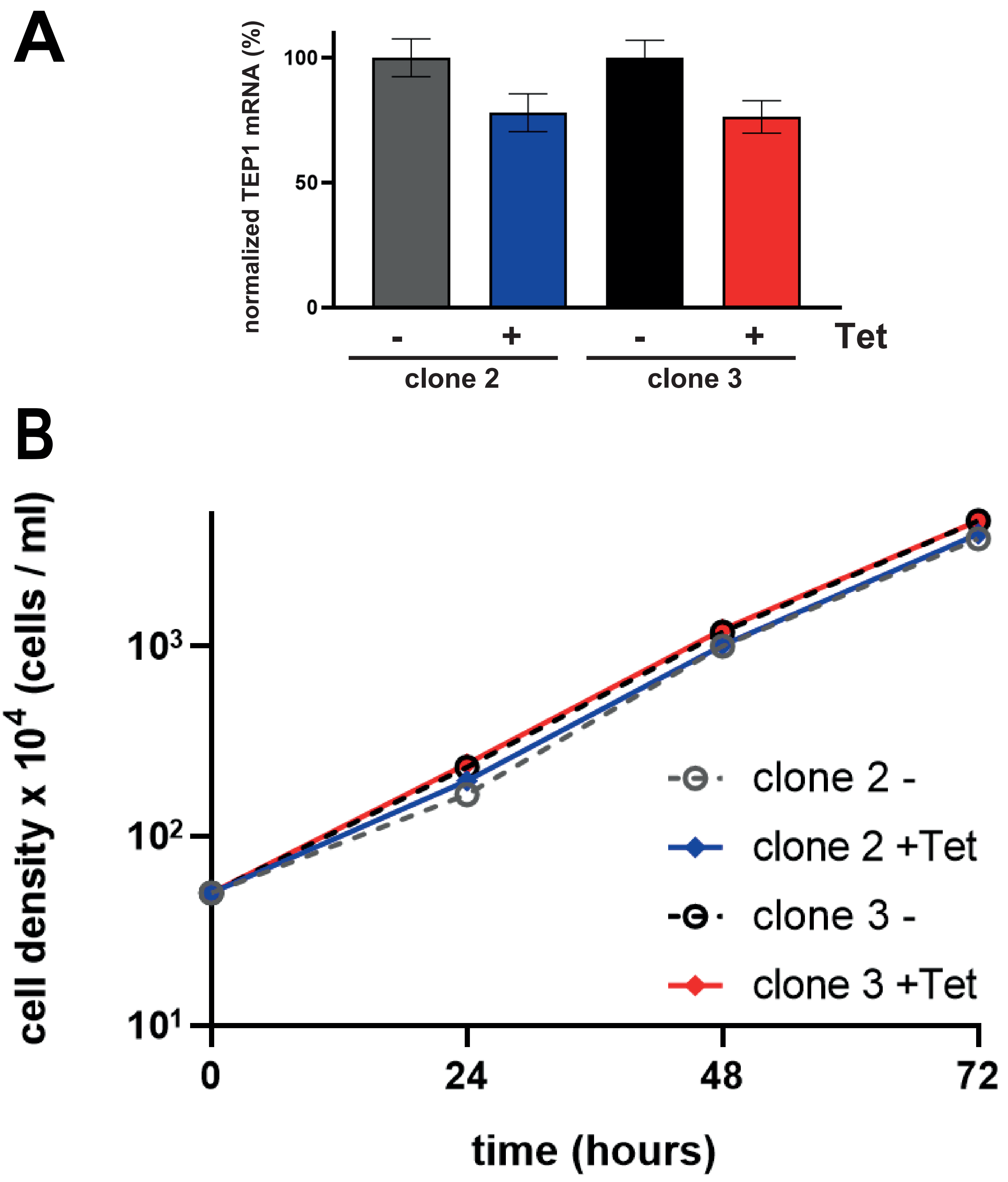

Figure S13. TEP1 RNAi. (A) MVP1 was depleted by inducible stem-loop RNAi which was confirmed by quantitative RT-PCR. A bar graph shows the level of TEP1 mRNA in the presence and absence of tetracyclin (Tet) for two clonal cell lines normalized using quantification of glyceraldehyde 3-phosphate dehydrogenase (GAPDH) mRNA (for raw data see Table S4). (B) Corresponding growth curve were recorded over a 72 h time period.
