# Supplementary material for: The trypanosome vault particle is composed of multiple major vault protein paralogs and harbors vault RNA
Source: J Biol Chem. 2025 Sep 11;301(10):110706. doi: 10.1016/j.jbc.2025.110706 (PMC12547018; doi:10.1016/j.jbc.2025.110706)
Supplement: Supporting Figure S14 [file mmc19.pdf]

Figure S14

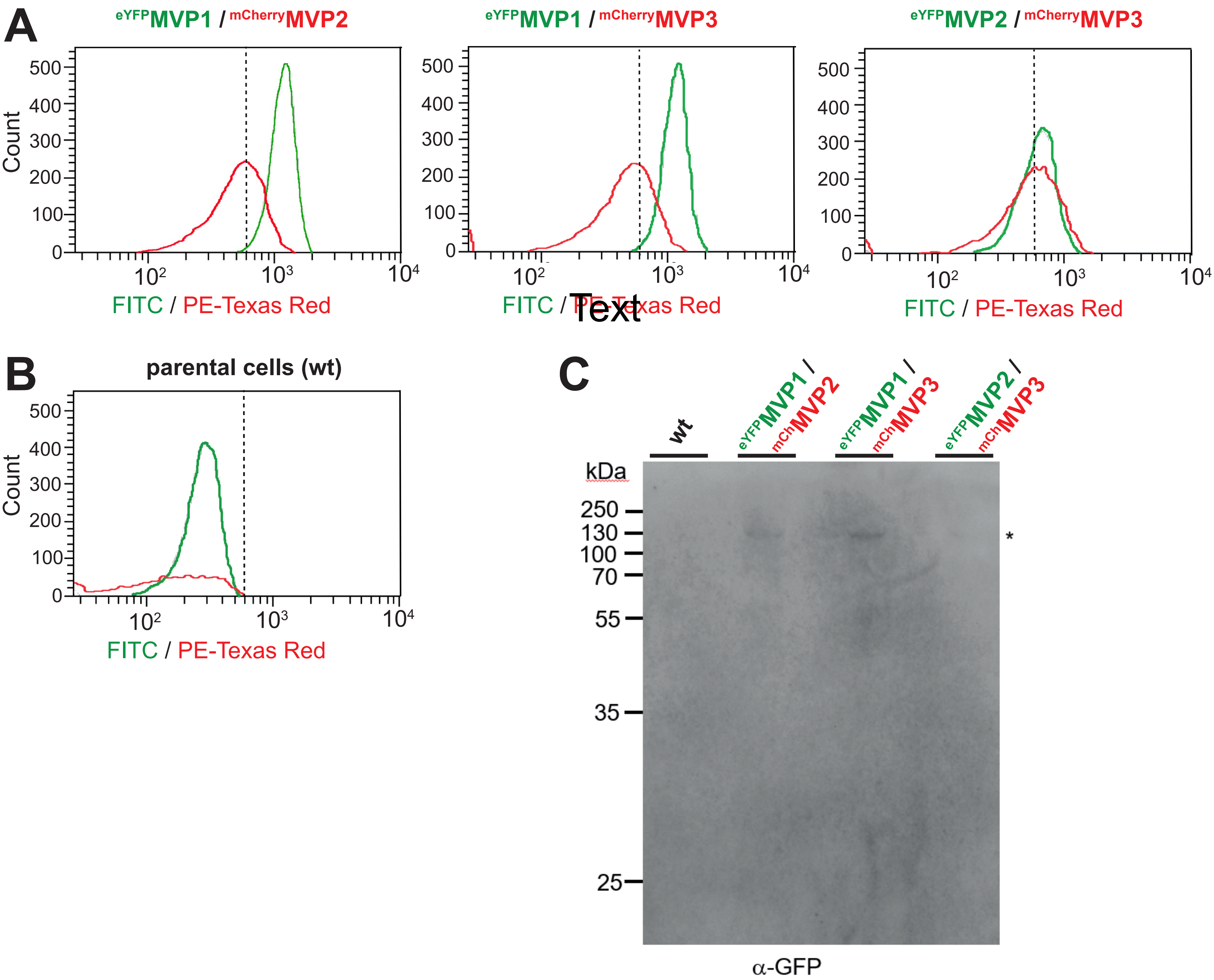

FigureS14. Flow cytometry and Western blot analyses of double tagged cell lines. Clonal cell lines endogenously expressing all combinations of MVPs, with one fused to eYFP and the other to mCherry were subjected to flow cytometry analyses. All three double-tagged lines show increased fluorescence at both wavelengths (A) in comparison to a wt control (B), proving expression of both GFP variants. These MVP fusions were detected by western blotting with anti-GFP antibody with a migration consistent with their molecular weight of (approximately 130 kDa; asterisk). Note that the three MVP paralogs are indistinguishable in SDS- PAGE due to their near identical molecular weight.
