# Supplementary material for: The trypanosome vault particle is composed of multiple major vault protein paralogs and harbors vault RNA
Source: J Biol Chem. 2025 Sep 11;301(10):110706. doi: 10.1016/j.jbc.2025.110706 (PMC12547018; doi:10.1016/j.jbc.2025.110706)
Supplement: Supporting Figure S15 [file mmc20.pdf]

Figure S15

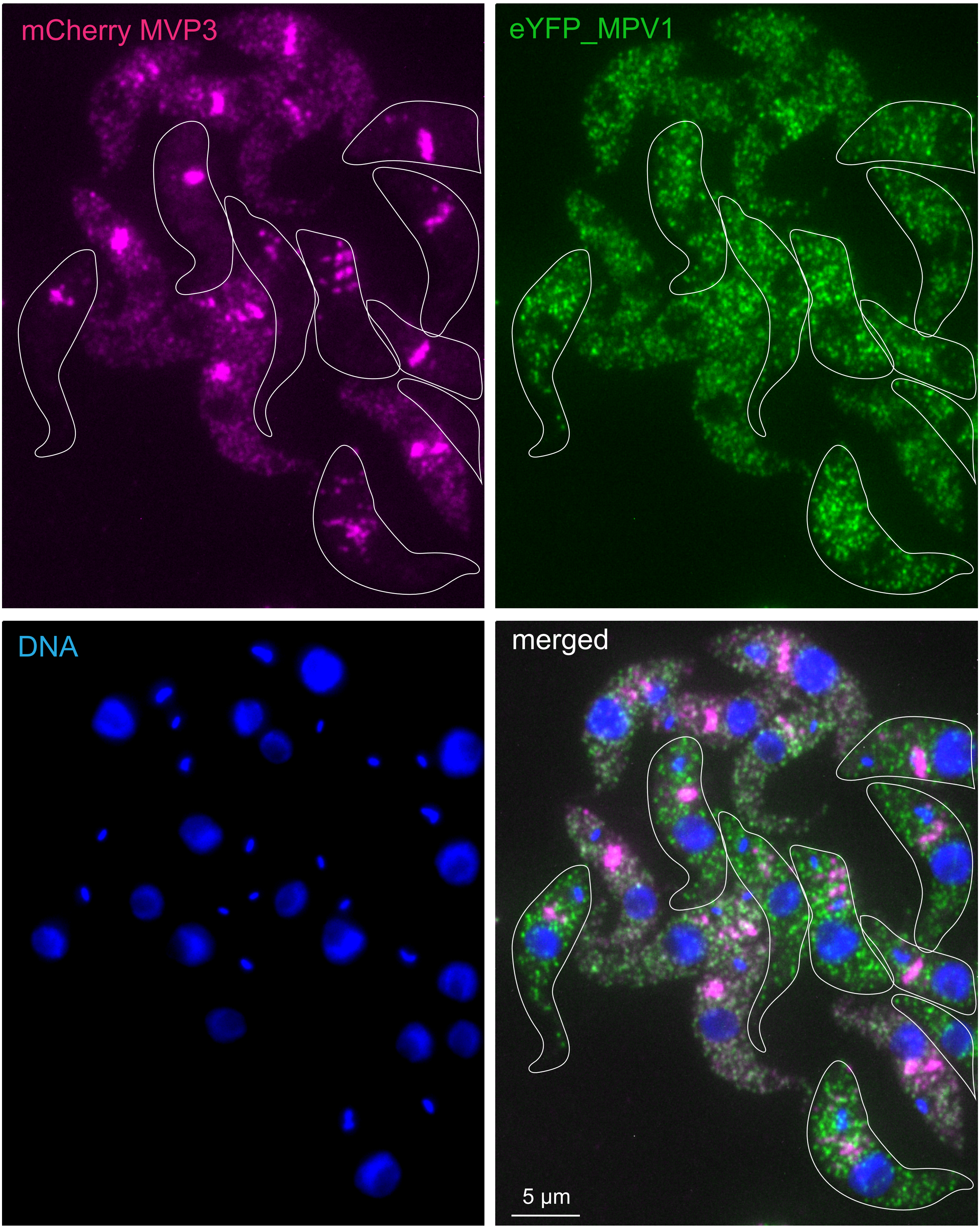

Figure S15. The vault shell is composed of all three MVP paralogs. MVP paralogs (MVP1=Tb927.5.4460 and MVP3=Tb927.10.6310) were endogenously tagged in *T. brucei* PCF with eYFP (MVP1) and mCherry (MVP3) at the N-terminus. Shown are single plane r aw images for eYFP (green) and mCherry (magenta) and DAPI (blue) of a 1:1 mixture of eYFP_MVP1 cells and eYFP_MVP1/mCherry_MVP3 double tagged cells, and a respective merge. Autofluorescence background signals arising from the lysosome and late endosomal compartments in the red channel. However, as the endocytic system of *T. brucei* is entirely restricted to the part posterior of the nucleus, the anterior cell part remains unaffected by the background signal and thus can be used for colocalization-studies (see Figure 6).
