# Supplementary material for: The trypanosome vault particle is composed of multiple major vault protein paralogs and harbors vault RNA
Source: J Biol Chem. 2025 Sep 11;301(10):110706. doi: 10.1016/j.jbc.2025.110706 (PMC12547018; doi:10.1016/j.jbc.2025.110706)
Supplement: Supporting Figure S16 [file mmc21.pdf]

Figure S16

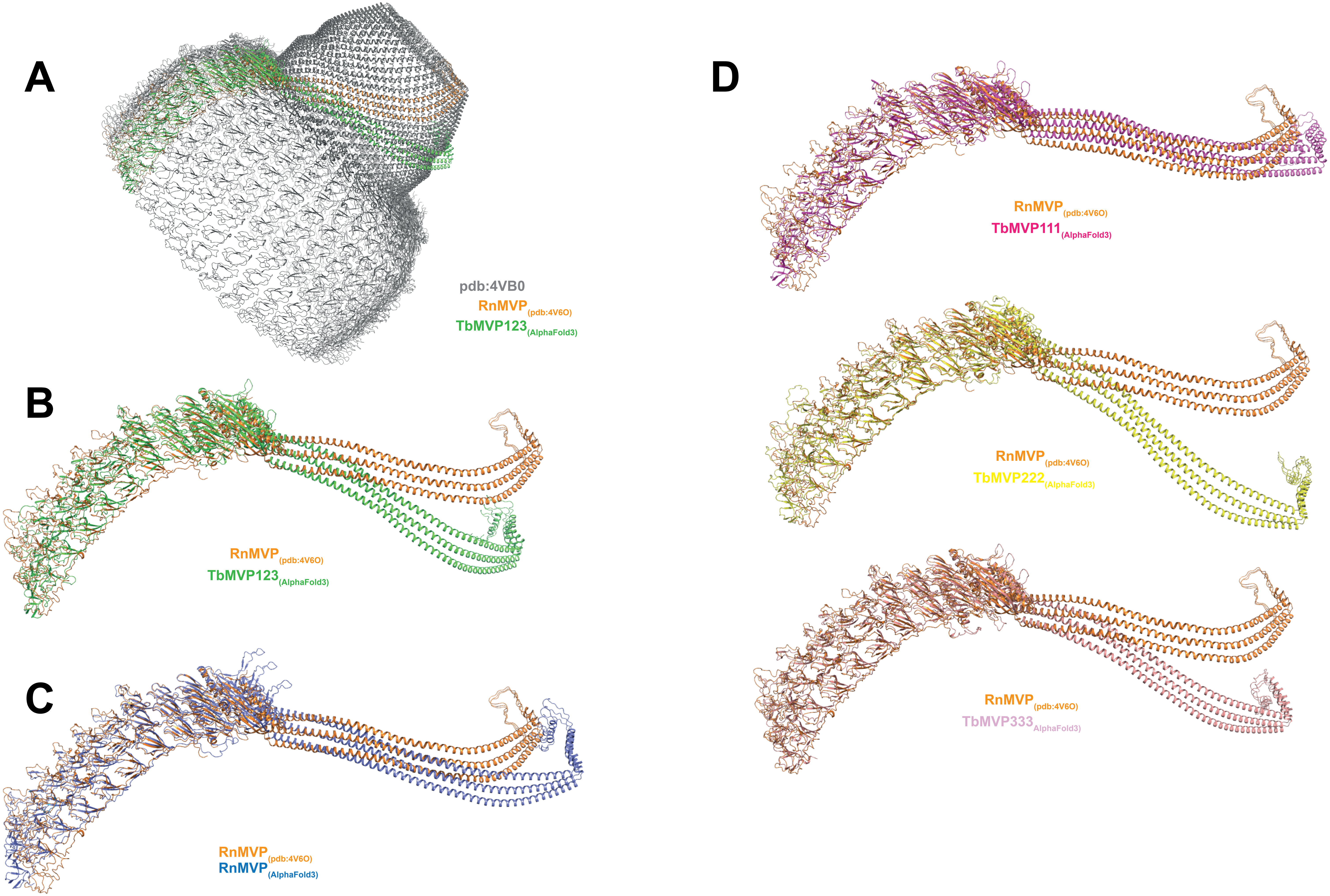

Figure S16: Alphafold3 models of trimeric MVP assemblies. Trimeric MVP assemblies were modeled with Alphafold3 [50]. (A, B) The heterotrimeric *T. brucei* MVP model (TbMVP123; green cartoon depiction) exhibits high structural similarity with mammalian MVP (pdb:4V60; orange) as visualized by structural alignment. The position of the cap-helix relative to the repeat-domains, dependent on the bent in the shoulder domain, is different. (C) This is also the case when comparing an Alphafold model of trimeric rat MVP with the experimental structure (bottom). (D) Homotrimeric *T. brucei* MVP models (MVP111, MVP222, MVP333) adopt similar structures with variations of the shoulder domain bent.
