# Supplementary material for: The trypanosome vault particle is composed of multiple major vault protein paralogs and harbors vault RNA
Source: J Biol Chem. 2025 Sep 11;301(10):110706. doi: 10.1016/j.jbc.2025.110706 (PMC12547018; doi:10.1016/j.jbc.2025.110706)
Supplement: Supporting Figure S17 [file mmc22.pdf]

Figure S17

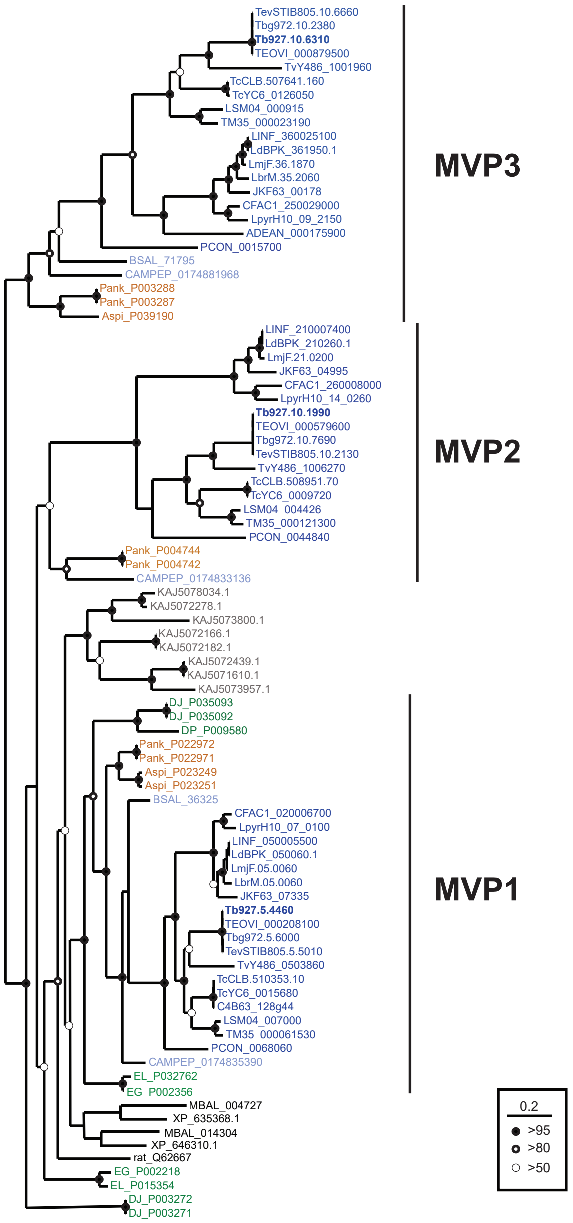

Figure S17: Phylogenetic tree of Discoba and Metamonada MVP sequences. Selected organisms from Trypanosomatida (dark blue), Bodonids (light blue), Prokinetoplastida (orange). All sequences used are detailed and annotated in Table S3. Support values for nodes exhibiting >50% bootstrap support or better are indicated symbolically (see inset). Sequences of mammalian (*Rattus norvegicus*) and Amoebozoa (*Dictyostelium discoideum, Mastigamoeba balamuthi*) MVP (black) were included, as previous key studies were mainly performed in these phylae.
